# Tumor response and endogenous immune reactivity after administration of HER2 CAR T cells in a child with metastatic rhabdomyosarcoma

**DOI:** 10.1038/s41467-020-17175-8

**Published:** 2020-07-15

**Authors:** Meenakshi Hegde, Sujith K. Joseph, Farzana Pashankar, Christopher DeRenzo, Khaled Sanber, Shoba Navai, Tiara T. Byrd, John Hicks, Mina L. Xu, Claudia Gerken, Mamta Kalra, Catherine Robertson, Huimin Zhang, Ankita Shree, Birju Mehta, Olga Dakhova, Vita S. Salsman, Bambi Grilley, Adrian Gee, Gianpietro Dotti, Helen E. Heslop, Malcolm K. Brenner, Winfried S. Wels, Stephen Gottschalk, Nabil Ahmed

**Affiliations:** 1 Texas Children’s Cancer and Hematology Centers, Texas Children’s Hospital, Baylor College of Medicine, Houston, TX USA; 2 Center for Cell and Gene Therapy, Texas Children’s Hospital, Houston Methodist Hospital, Baylor College of Medicine, Houston, TX USA; 3 Department of Pediatrics, Baylor College of Medicine, Houston, TX USA; 4 Department of Pediatrics, Yale University School of Medicine, New Haven, CT USA; 5 Department of Medicine, Baylor College of Medicine, Houston, TX USA; 6 Department of Pathology and Immunology, Baylor College of Medicine, Houston, TX USA; 7 Department of Pathology, Yale University School of Medicine, New Haven, CT USA; 8 Department of Microbiology and Immunology at University of North Carolina, Chapel Hill, NC USA; 9 Lineberger Cancer Center at University of North Carolina, Chapel Hill, NC USA; 10 Georg-Speyer-Haus, Institute for Tumor Biology and Experimental Therapy, Frankfurt, Germany; 11 German Cancer Consortium (DKTK), Partner Site Frankfurt/Mainz, Frankfurt, Germany; 12 Frankfurt Cancer Institute, Goethe University, Frankfurt, Germany

**Keywords:** Cancer, Immunology, Oncology, Sarcoma

## Abstract

Refractory metastatic rhabdomyosarcoma is largely incurable. Here we analyze the response of a child with refractory bone marrow metastatic rhabdomyosarcoma to autologous HER2 CAR T cells. Three cycles of HER2 CAR T cells given after lymphodepleting chemotherapy induces remission which is consolidated with four more CAR T-cell infusions without lymphodepletion. Longitudinal immune-monitoring reveals remodeling of the T-cell receptor repertoire with immunodominant clones and serum autoantibodies reactive to oncogenic signaling pathway proteins. The disease relapses in the bone marrow at six months off-therapy. A second remission is achieved after one cycle of lymphodepletion and HER2 CAR T cells. Response consolidation with additional CAR T-cell infusions includes pembrolizumab to improve their efficacy. The patient described here is a participant in an ongoing phase I trial (NCT00902044; active, not recruiting), and is 20 months off T-cell infusions with no detectable disease at the time of this report.

## Abstract (web summary)

Recurrent metastatic rhabdomyosarcoma remains largely incurable. Here, the authors describe a child with metastatic rhabdomyosarcoma who has durable response to HER2-specific CAR T cells and shows endogenous immune reactivity.

## Introduction

Rhabdomyosarcoma (RMS) is the most common malignant soft-tissue tumor in children and adolescents. There is no effective treatment for patients with metastatic RMS refractory to standard cytotoxic chemotherapy^1,2^. We have previously reported on a favorable safety profile of autologous HER2 CAR T cells in patients with advanced sarcoma whose tumors lack HER2 gene-amplification and are thus not amenable to HER2-specific monoclonal antibody therapy^3–5^. Although clinical benefit was achieved in a subset of patients after HER2 CAR T cells, with 4 of 17 evaluable patients having durable stable disease and 1 patient with partial response (PR), no measurable complete tumor response was observed^3^. A major limitation identified in the study was the lack of T-cell expansion and persistence in all treated patients. Lymphodepletion prior to T-cell administration improves the expansion of adoptively transferred T cells, in part due to increased availability of homeostatic cytokines such as IL-7 and IL-15. In addition, lymphodepleting chemotherapy decreases both regulatory T cells and myeloid-derived suppressor cells, thereby augmenting the expansion of T cells and consequently their antitumor activity^6–10^.

An ongoing phase I trial (NCT00902044; active, not recruiting) is designed to evaluate the safety of autologous HER2 CAR T cells after lymphodepletion in patients with advanced sarcoma and incorporates multiple CAR T-cell infusions to improve their persistence. Here, we report on an exceptional tumor response observed in a child with refractory bone marrow-metastatic RMS enrolled on this phase I trial. Furthermore, we describe the longitudinal analysis of immune-monitoring studies and show evidence of endogenous immune reactivity accompanying CAR T-cell therapy, which may have contributed to this favorable clinical outcome^11^.

## Results

### Clinical history and infusion of autologous HER2 CAR T cells

**Fig. 1:**
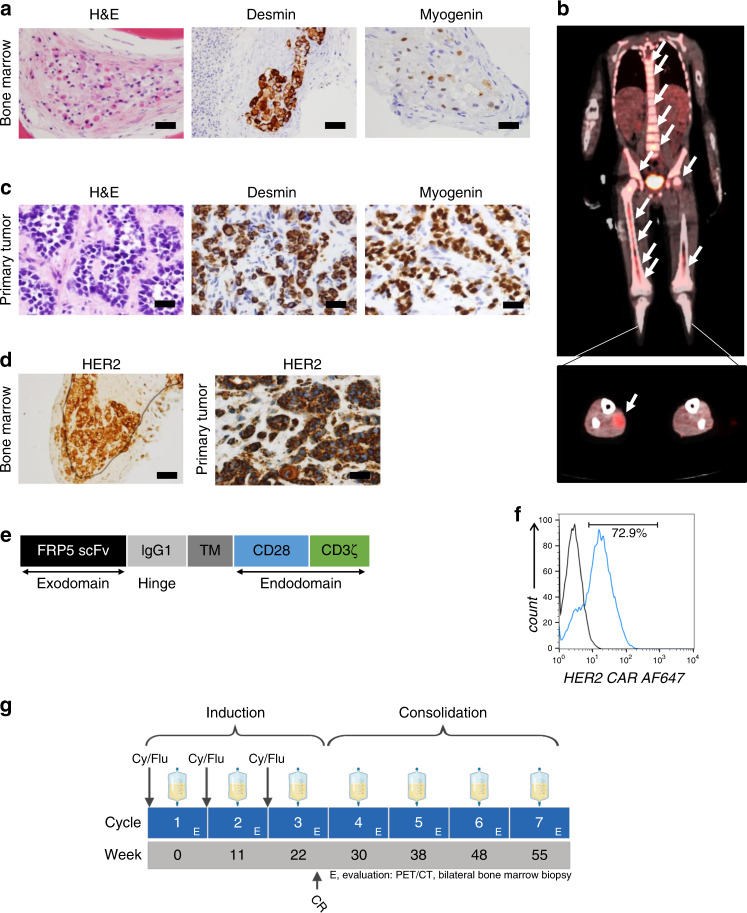
Clinical and pathological findings prior to enrollment, and the CAR T-cell infusion regimen. **a** Histological examination showing hypocellular bone marrow (BM) containing alveolar-patterned rhabdomyosarcoma (RMS) cells on routine hematoxylin and eosin (H&E) staining and immunoreactivity to desmin and myogenin. **b** Positron emission tomography–computed tomography showing extensive BM involvement (upper panel) and a primary tumor in the right calf (lower panel). **c** Histological examination of the primary tumor showing RMS cells that are immunoreactive to desmin and myogenin. **d** HER2 immunoreactivity (grade 3, intensity score 3+) of the primary tumor and BM metastasis at baseline prior to study enrollment. Panels (**a**, **c**, **d**) show representative microscopic images; scale bar 100 µm. **e** Schematic outline of components of the HER2 CAR transgene introduced by retroviral vector transduction. TM transmembrane. **f** HER2 CAR expression in the autologous T-cell product released from the good manufacturing practice (GMP) laboratories for infusion. **g** Treatment regimen, including the induction and consolidation phases. Autologous HER2 CAR T-cell dose was 1 × 10^8^ cells/m^2^ for each infusion. H&E hematoxylin and eosin stain. Cy/Flu cyclophosphamide and fludarabine.

A 7-year-old boy presenting with severe pancytopenia was diagnosed with metastatic RMS after bilateral bone marrow aspiration and biopsy (BMAB) revealed infiltration with alveolar-pattern RMS cells, which were immunoreactive to desmin and myogenin (Fig. 1a). A whole-body positron emission tomography–computed tomography (PET-CT) revealed extensive BM involvement and a primary tumor in the right calf muscle (Fig. 1b). Surgical resection of the primary tumor confirmed the diagnosis of RMS with alveolar histology (Fig. 1c) but without evidence of *FOXO1*-gene rearrangement. Tumor DNA sequencing detected a somatic variant of *PIK3CA Q546R*. The child completed intense systemic chemotherapy lasting for 13 months and radiation therapy to the primary site (4140 cGy in 23 fractions) according to the Children’s Oncology Group’s (COG) co-operative trial ARST0431 for upfront treatment of high-risk RMS (Supplementary Table 1), resulting in local control^12^. After 9 months of chemotherapy, the PET-CT showed no FDG-avid disease, but at the conclusion of treatment, BMAB showed ~10% of the cellularity comprised of metastatic RMS. By the specific protocol criteria, the child was considered to have PR at the end of first-line therapy and subsequently received salvage therapy per the COG protocol ARST0921 (Supplementary Table 1)^13^. Restaging evaluation after two cycles of chemotherapy showed persistent metastatic RMS aggregates comprised of atypical cells with abundant pale eosinophilic cytoplasm and large eccentric, irregular nuclei with inconspicuous nucleoli in the bone marrow. Because of the chemotherapy-refractory metastatic disease, the child was evaluated for phase I trials. After confirming HER2 surface expression on both the primary tumor and BM metastasis by immunohistochemistry (IHC; grade 3, intensity score 3; Fig. 1d)^14^, the child was enrolled on our phase I trial of autologous HER2 CAR T cells in patients with advanced sarcoma. An autologous HER2 CAR T-cell product was manufactured and contained a majority of CD8+ T cells with an effector memory profile (CD45RO+/CCR7−/CD62L−; Supplementary Fig. 1a). The FRP5 antibody-based HER2-specific CAR molecule containing a CD28 co-stimulatory endo-domain (Fig. 1e) was detected in 72.9% of the patient’s T cells (Fig. 1f; gating strategy shown in Supplementary Fig. 2) and induced specific lysis of the HER2-positive sarcoma cells LM7 but not of the HER2-negative cell line K562 (Supplementary Fig. 1b)^5^. After a 4-week washout period and recovery from prior cytotoxic chemotherapy, the child received three intravenous infusions of 1 × 10^8^ cells/m^2^ autologous HER2 CAR T cells ~10 weeks apart, each after lymphodepletion with cyclophosphamide and fludarabine (Cy/Flu)^6,7^. At the end of this induction phase, no morphologic or imaging evidence of RMS was present. Thereafter, the same dose of HER2 CAR T cells was administered approximately every 10 weeks without Cy/Flu for 6 additional months with the intent of consolidating the disease response (Fig. 1g).

### HER2 CAR T cells expand safely after lymphodepletion

**Fig. 2:**
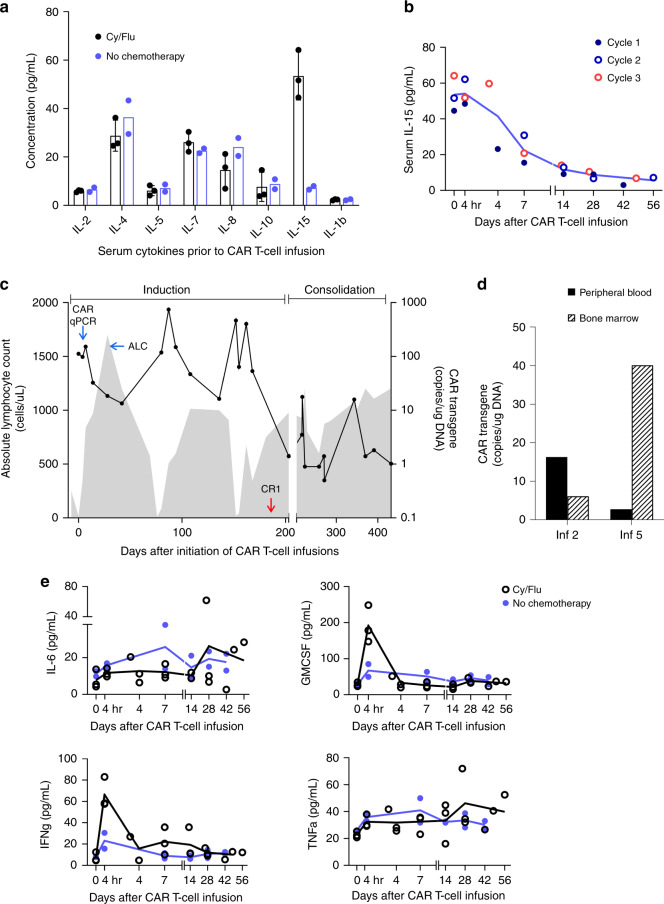
Measurement of serum cytokines and monitoring of HER2 CAR T cells after infusion. **a** Analysis of serum cytokines after Cy/Flu administration and prior to T-cell infusion on day 0 showing the difference in IL-15 levels with (*n* = 3 infusion cycles, data presented as mean values ± standard deviation) and without (*n* = 2 infusions) lymphodepletion. **b** Kinetics of serum IL-15 levels prior to and after T-cell infusions given with cytoreducing chemotherapy (*n* = 3 infusion cycles). **c** Trends in the absolute lymphocyte count (ALC; shaded gray area) and levels of the HER2 CAR transgene detected by quantitative polymerase chain reaction (qPCR; solid black line) in the peripheral blood during the induction and consolidation phase leading to the initial complete response (CR1). **d** Detection of the HER2 CAR transgene in the peripheral blood and corresponding bone marrow levels at 6 weeks after infusions 2 and 5. **e** Analysis of pro-inflammatory cytokines (IL-6, GM-CSF, IFNγ, and TNFα) in the patient’s serum before and after CAR T-cell infusion given with (*n* = 3 infusion cycles) and without (*n* = 2 infusions) lymphodepletion. In panels (**a**, **b**, **e**), each dot in the graph represents an average of technical replicates from a biologically distinct serum sample. Inf CAR T-cell infusion, Cy/Flu cyclophosphamide and fludarabine.

The Cy/Flu regimen induced grade 4 lymphopenia, with an absolute lymphocyte count of 11–19 cells/mm^3^ on the day of HER2 CAR T-cell infusion (day 0). Analysis of the child’s serum after conditioning chemotherapy but prior to T-cell infusions revealed an increase in the homeostatic cytokine IL-15, with peak levels on the day of T-cell infusion that declined to low but detectable levels by 6 weeks post-infusion (Fig. 2a, b)^10^. HER2 CAR T cells were detected in the peripheral blood using quantitative polymerase chain reaction (qPCR) starting at 3 h and up to 8 weeks after each infusion during induction, with the peak in vivo expansion (152–747 copies/μg of DNA) occurring 7 days post infusion (Fig. 2c). Matched 6-week post infusion BMAB samples, when available, were analyzed for the presence of the HER2 CAR transgene (Fig. 2d).

During induction, the child developed grade 1 cytokine release syndrome (CRS) with fever, malaise, chills, and nausea within 12 h of each infusion, which resolved completely with supportive care within 72 h of onset (other adverse events are detailed in Supplementary Table 2)^15^. We detected a rise in C-reactive protein (range: 3.9–8.4 mg/dL) within 24 h of T-cell infusions given after Cy/Flu that trended down with resolution of the fever. GM-CSF and IFN-γ were elevated in the peripheral blood at 4 h post infusion when T cells were administered after lymphodepletion, and a small but sustained increase in the serum IFN-γ (but not IL-6 or TNFα) was noted between days 7–14, which corresponded to the peak of CAR T-cell expansion (Fig. 2e). In addition to lymphopenia, the child developed grades 3–4 neutropenia (absolute neutrophil count: 180–747 cells/mm^3^) lasting up to day 14 with each Cy/Flu conditioning during induction. No cardiac or pulmonary toxicities were observed despite the CAR T-cell expansion achieved and the repeated infusions given. Echocardiograms performed 6 weeks after each HER2 CAR T-cell infusion showed normal left ventricular ejection fraction.

### Exceptional tumor response to HER2 CAR T cells

**Fig. 3:**
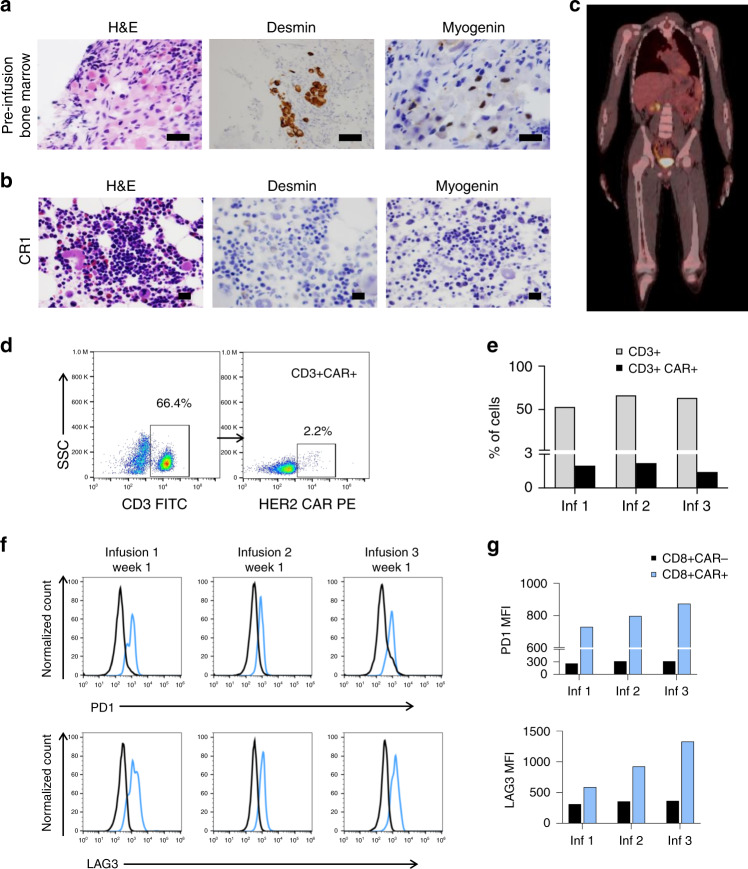
Clinical and pathological findings after autologous HER2 CAR T-cell infusions. **a** Histological examination of the bone marrow 4 weeks after the salvage chemotherapy (ARST0921) and prior to initiating CAR T-cell infusions showing hypocellularity and presence of rhabdomyosarcoma (RMS) cells on hematoxylin and eosin (H&E) staining and immunoreactivity to desmin and myogenin, **b** complete disease response (CR1), evidenced by recovery of trilineage hematopoiesis and absence of immunoreactivity to desmin and myogenin after three HER2 CAR T-cell infusions. Panels (**a**, **b**) show representative microscopic images; scale bar 100 µm. **c** Representative image from positron emission tomography–computed tomography (PET-CT) with no evidence of FDG-avid disease in bone marrow or other sites 6 weeks after the third HER2 CAR T-cell infusion. **d** Detection of HER2 CAR-expressing T cells in the peripheral blood 7 days after the second infusion using flow cytometry. HER2 CAR was specifically recognized using HER2.Fc chimeric protein followed by a goat anti-human Fc conjugated with PE as a secondary antibody. SSC side scatter. **e** The proportion of CD3+ HER2 CAR-expressing T cells on day +7 after each infusion during the induction period. **f** Histograms showing the PD-1 and LAG3 surface expression in CAR-positive CD8+ (in blue) in comparison to CAR-negative CD8+ T cells (in black) at peak expansion (day +7) after each infusion during induction, and **g** the corresponding median fluorescence intensity (MFI) of PD-1 and LAG3 surface expression in CAR-positive and CAR-negative CD8+ T cells.

Bilateral BMABs, standard of care for the assessment of BM metastatic RMS, were obtained prior to initiation of study treatment (Fig. 3a) and 6 weeks after each CAR T-cell infusion. Stable disease was observed following the first and the second CAR T-cell infusions with 10–20% of the bone marrow cellularity comprised of metastatic tumor aggregates. The BMAB following the third CAR T-cell infusion demonstrated recovery of trilineage hematopoiesis and no morphological evidence of RMS cells (Fig. 3b). The whole-body PET-CT remained negative with no evidence of FDG-avid disease (Fig. 3c). The child was considered to have a complete response (CR) as per the study-specific criteria. CAR T cells were detected in the peripheral blood by flow cytometry only during peak expansion (day 7) and constituted 1.5–2.2% of the T lymphocytes (Fig. 3d, e; gating strategy shown in Supplementary Fig. 3). Further analysis of the CD8+ T-cell subset revealed higher intensity of surface expression of PD-1 and LAG3 in CAR-positive T cells compared to CAR-negative T cells (Fig. 3f, g; gating strategy shown in Supplementary Fig. 4). Subsequently, the child received four additional CAR T-cell infusions ~10 weeks apart without lymphodepletion over 6 months, with the intent of consolidating the tumor response. To account for the potential sampling error from patchy involvement of the bone marrow, bilateral BMAB and PET-CT were done 6 weeks after each T-cell infusion throughout the treatment period and every 12 weeks during the follow-up. These evaluations continued to show no evidence of disease, and the response was sustained for 12 months.

### Remodeling of T-cell receptor repertoire after CAR T-cell therapy

**Fig. 4:**
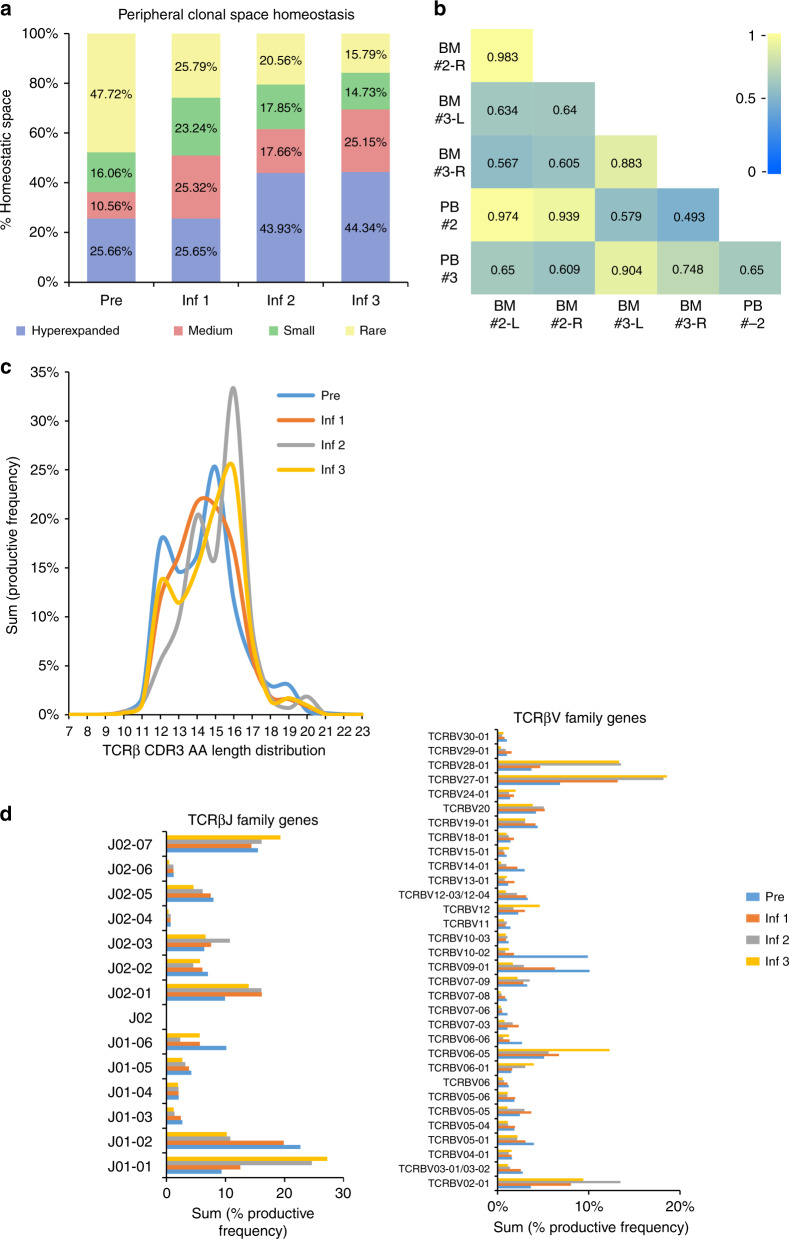
Remodeling of TCRβ repertoire following HER2 CAR T-cell infusions. **a** Longitudinal homeostatic space distribution of T-cell clones from the peripheral blood (PB) categorized as hyperexpanded/large (>1% frequency of productive rearrangements), medium (0.1–1% frequency), small (more than single event, but <0.1% frequency) and rare (single rearrangement events) before and 6 weeks after the first, second and third HER2 CAR T-cell infusions. The pre-infusion sample (Pre) was obtained at 4 weeks from the prior cyclophosphamide containing chemotherapy and serves as a chemotherapy only control. **b** Heat map representing Morisita’s overlap index of TCRβ CDR3 rearrangements between time-matched samples from the PB and bone marrow (BM) obtained 6 weeks after the second and third CAR T-cell infusions. The overlap index has values ranging from 0 to 1 representing low to high degree of overlap, respectively. **c** Amino acid (AA) length distribution of the TCRβ CDR3 in peripheral blood before and 6 weeks after the first, second and third HER2 CAR T-cell infusions. **d** TCRβV family genes and TCRβJ family genes in the CDR3 region of peripheral blood T cells before and 6 weeks after the first, second and third HER2 CAR T-cell infusions. Only TCRβV genes with >1% of cumulative productive frequencies are represented. Complete TCRβV family gene use is provided as Supplementary Table 4. Inf infusion, L left, R right.

Resolution of the metastatic disease was achieved in this child after the initial disease stabilization despite heterogeneous HER2 expression. To evaluate whether CAR T cells initiated an endogenous immune response that could have potentially contributed to tumor control, we carried out a longitudinal sequencing of the CDR3 region of TCRβ in the peripheral blood mononuclear cells before and after the initiation of CAR T-cell therapy^16^. The pre-infusion peripheral blood sample was obtained at study entry, 4 weeks after recovering from the prior chemotherapy. The post-infusion peripheral blood and the time-matched bone marrow aspiration samples representing the metastatic disease sites were analyzed at 6 weeks after the CAR T-cell infusions. During the course of induction, T cells represented an increasing proportion of the peripheral blood mononuclear cells (Supplementary Fig. 5a) comprised more of oligoclonally expanded populations after the second and third CAR T-cell infusions (sample clonality 0.332 and 0.331, respectively), compared to pre-infusion baseline (sample clonality 0.19; Supplementary Table 3). Six weeks after the second and third CAR T-cell infusions, the immunodominant clonotypes (hyperexpanded clones, defined as having >1% rearrangement frequency) occupied more of the peripheral blood clonal homeostatic space (43.93% and 44.34%, respectively) as compared to 25.66% at the pre-infusion time point (Fig. 4a). The T cells in the peripheral blood also had a high degree of clonal similarity with the time-matched bone marrow samples (Fig. 4b and Supplementary Fig. 5b), and the distribution of the clonal space homeostasis was comparable between the left and the right bone marrow metastatic disease sites (Supplementary Fig. 5c). Analysis of the in-frame CDR3 loop amino acid sequence length to evaluate the patterns of TCR utilization demonstrated diversity in the peripheral repertoire between CAR T-cell infusions (Supplementary Table 4a). A marked perturbation in the CDR3 length distribution was observed after the second and third infusions (Fig. 4c), further suggesting a differential expansion of unique TCRβ clonotypes^17^. The CDR3 length distribution patterns are primarily determined by the variable-region gene assembly^18^, and in this child, we found distinct alterations in the TCRβV family gene usage after CAR T-cell infusions (predominantly TCRβV02, TCRβV06, TCRβV27, and TCRβV28) in comparison to pre-infusion (predominantly TCRβV09 and TCRβV10) peripheral blood (Fig. 4d and Supplementary Table 4b). Similarly, utilization of TCRβJ family genes varied between pre- (predominantly TCRβJ01-02) and post-CAR T-cell infusion (predominantly TCRβJ01-01 and TCRβJ02-01) peripheral blood T cells (Fig. 4d and Supplementary Table 4c).

**Fig. 5:**
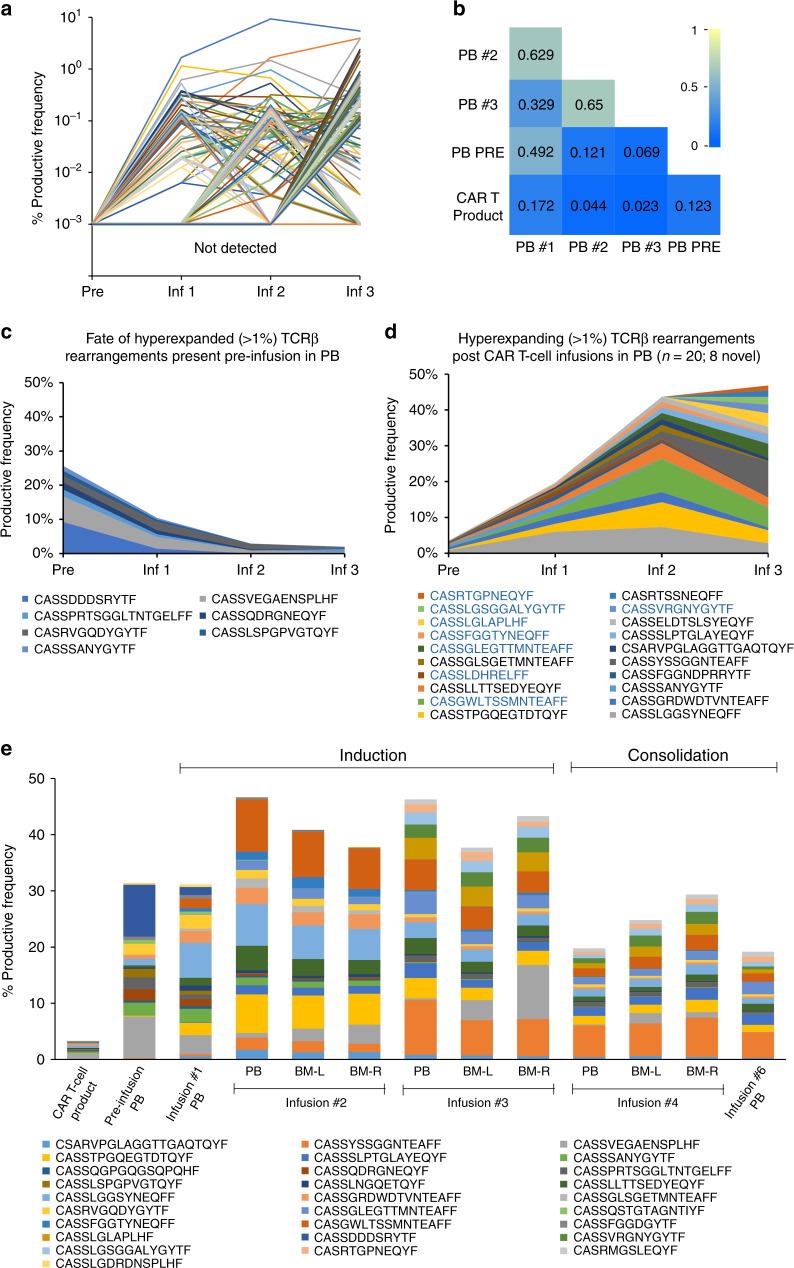
Longitudinal tracking of productive TCRβ CDR3 rearrangements. **a** Fate of TCRβ CDR3 rearrangements which developed after the initiation of HER2 CAR T-cell infusions, from top 250 rearrangements (*n* = 127). **b** Heat map representing Morisita’s overlap index of TCRβ rearrangements between the infused CAR T-cell product and longitudinal samples from the peripheral blood demonstrating restructuring of the T-cell repertoire with each CAR T-cell infusion. The overlap index has values ranging from 0 to 1 depicting low to high degree of overlap, respectively. **c** Hyperexpanded (defined as having >1% frequency) TCRβ CDR3 rearrangements present in the peripheral blood prior to initiation of CAR T-cell infusions and their fate over the course of induction. **d** Longitudinal tracking of the productive TCRβ CDR3 rearrangements expanding in the peripheral blood analyzed 6 weeks after HER2 CAR T-cell infusions given during induction phase. Eight T-cell clones that were not detected pre-infusion or in the infused T-cell product were detected in the peripheral blood following CAR T-cell infusions (highlighted in blue). **e** Frequency distribution of top 10 TCRβ CDR3 rearrangements present in each peripheral blood (PB) and bone marrow (BM) sample 6 weeks after the T-cell infusion, with (induction) or without (consolidation) Cy/Flu lymphodepletion, in comparison to that of pre-infusion peripheral blood and the infused CAR T-cell product. Pre-infusion peripheral blood was obtained at study entry, 4 weeks after the prior cyclophosphamide containing chemotherapy. BM samples were unavailable for analysis after infusions 1 and 6. Pre pre-infusion, Inf infusion, Cy/Flu cyclophosphamide and fludarabine, L left, R right.

The appearance of unique productive TCRβ rearrangements in the peripheral blood after each CAR T-cell infusion during induction (Fig. 5a and Supplementary Fig. 5d) suggests remodeling of the T-cell repertoire (Fig. 5b), primarily consisting of high frequency clones (Supplementary Fig. 5e). Upon longitudinal tracking of the productive TCRβ rearrangements, we noted an overall declining trend in the immunodominant clones that were present prior to the initiation of CAR T-cell infusions (Fig. 5c and Supplementary Table 5a). While a few pre-existing clones (detected in the pre-infusion sample) expanded during the course of treatment, of the 20 immunodominant clones identified, 8 were not detectable in the peripheral blood prior to initiation of CAR T-cell infusions or in the infused CAR T-cell product (Fig. 5d and Supplementary Table 5b). Comparing the frequency of distribution of the top 10 CDR3 rearrangements identified in each sample demonstrated progressive TCRβ repertoire remodeling after CAR T-cell infusions during induction (with Cy/Flu) that persisted through consolidation (without Cy/Flu) at a lower frequency (Fig. 5e). These repertoire patterns were distinct from that of pre-infusion peripheral blood and of the infused CAR T-cell product. The productive CDR3 rearrangements and the frequency of their distribution were largely comparable between the peripheral blood and the time-matched bone marrow samples.

### Antibody responses against oncogenic pathway proteins

**Fig. 6:**
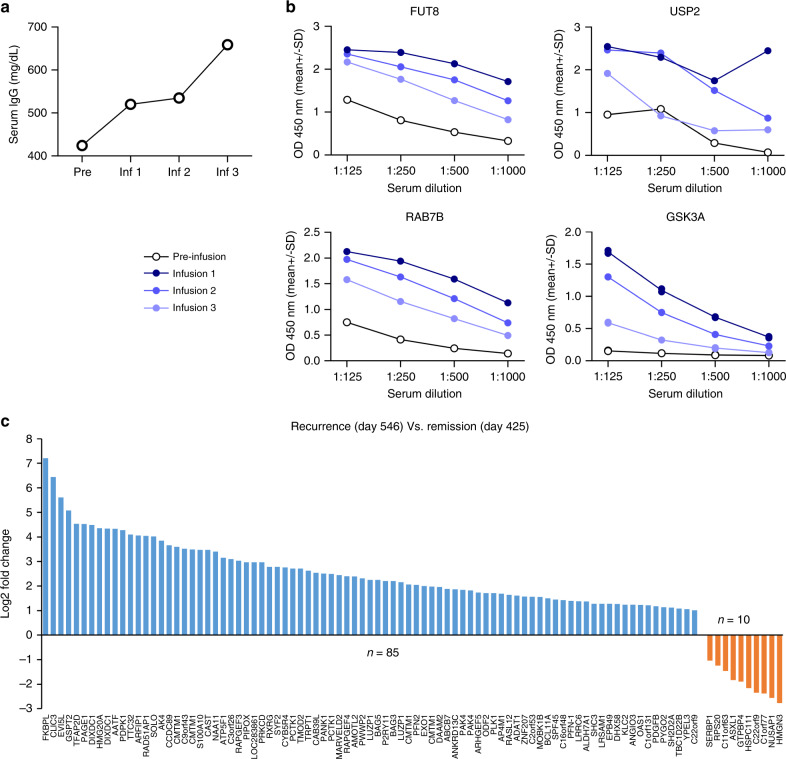
Autoantibody responses identified in the patient’s serum before and after CAR T-cell infusions. **a** Serum IgG levels obtained in the clinical laboratory prior to initiation of CAR T-cell infusion and 10 weeks after each infusion during the first induction period. **b** Indirect ELISA confirming serum antibody reactivity with recombinant FUT8, USP2, RAB7B, and GSK3A post HER2 CAR T-cell infusions during the first induction, in comparison to pre-infusion sample. Serum samples from each time point were tested in four dilutions as shown. **c** Waterfall plot depicting proteins with ≥2 fold change in autoantibody binding signal identified by ProtoArray^TM^ Human Protein Microarray analysis in the patient’s serum at tumor recurrence 6 months after stopping T-cell infusions (day 546) compared to the sample obtained during remission (day 425). Pre pre-infusion, Inf infusion.

Due to the intense lymphodepleting chemotherapy administered prior to CAR T-cell infusions, we monitored the immunoglobulin (IgG) levels in this child which paradoxically showed an increasing trend during induction (Fig. 6a). We then examined the child’s serum using ProtoArray™ proteomic analysis and identified specific post-infusion autoantibody responses that were largely directed against intracellular components involved in cell-cycle regulation, cell-signaling, and tumor-associated processes (Supplementary Fig. 6a and Supplementary Table 6)^19^. In particular, autoantibodies against four proteins (including FUT8, USP2, SULT1B1, and RAB7B), implicated in tumor invasion and metastasis^20^, were consistently high 6 weeks after the first, second, and third infusions (Supplementary Table 7). The reactivity of the child’s serum to FUT8, USP2, and RAB7B at different dilutions was validated using indirect ELISA (Fig. 6b). A marked post-infusion autoantibody response observed against GSK3α, a downstream target in the PI3K/AKT signaling pathway, was also confirmed using indirect ELISA (Fig. 6b)^21^. The serum autoantibody target profile at tumor recurrence (detailed below), 6 months after stopping CAR T-cell infusions (day 546), was noted to be distinct from that at remission (day 425; Fig. 6c and Supplementary Data 1), with a corresponding increase in the child’s serum IgG and IgM levels detected using indirect ELISA (Supplementary Fig. 6b).

### Bone marrow relapse and a sustained second remission

**Fig. 7:**
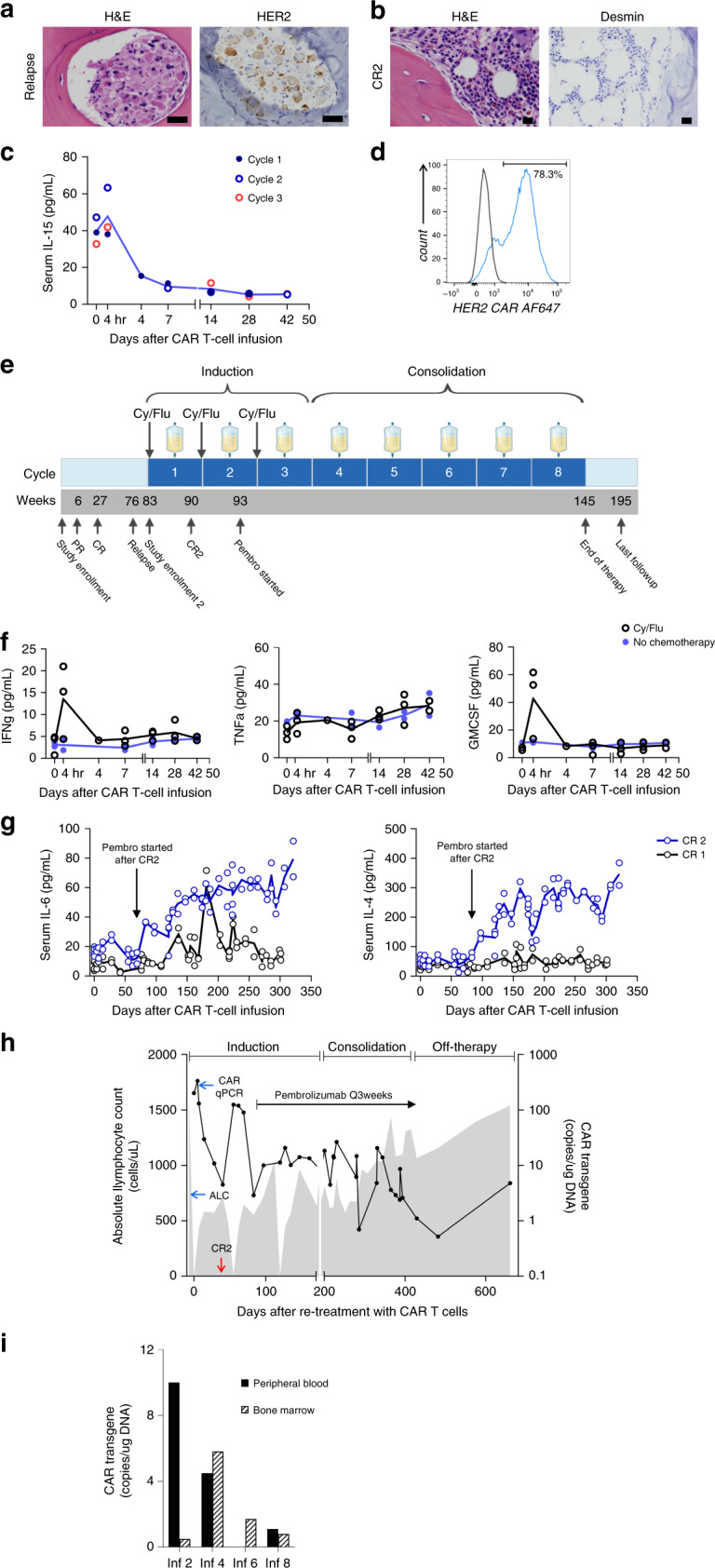
HER2 CAR T-cell infusions after bone marrow relapse and monitoring during the second remission. **a** Surveillance bone marrow (BM) at 6 months after stopping HER2 CAR T-cell infusions showing hypocellularity and presence of HER2-expressing RMS cells (grade 2, intensity score 2+ by immunohistochemistry). **b** BM showing restoration of trilineage hematopoiesis and no morphological evidence of RMS (CR2) 6 weeks after one cycle of lymphodepletion and HER2 CAR T cells (1 × 10^8^ cells/m^2^). Panels (**a**, **b**) show representative microscopic images; scale bar 100 µm. **c** Kinetics of serum IL-15 levels before and after HER2 CAR T-cell infusions given with Cy/Fly lymphodepletion (*n* = 3 infusion cycles). **d** HER2 CAR expression in the second autologous T-cell product manufactured and infused during consolidation of CR2. **e** Timeline of the initial treatment course, disease relapse, re-induction of CR2, and consolidation of the response shown in a schematic diagram. PD-1 antibody, pembrolizumab, was initiated 4 weeks after confirming the CR2 and continued every 3 weeks thereafter. **f** Analysis of pro-inflammatory cytokines (IFN-γ, TNFα, and GM-CSF) in the patient’s serum before and after CAR T-cell infusion given with (*n* = 3 infusion cycles) and without (*n* = 2 infusions) lymphodepletion. **g** Longitudinal monitoring of serum IL-6 and IL-4 levels during CR2, before and after adding PD-1-blocking antibody to the CAR T-cell infusion regimen, in comparison to CR1. Solid lines represent the mean values of sample duplicates tested. **h** Trends in the absolute lymphocyte count (ALC; shaded gray area) and levels of the HER2 CAR transgene detected by quantitative polymerase chain reaction (qPCR; solid black line) in the peripheral blood during the treatment phase of CR2 and the follow-up period. **i** Detection of HER2 CAR transgene in the matched BM and peripheral blood samples at 6 weeks after T-cell infusions during CR2. In panels (**c**, **f**), each dot in the graph represents an average of technical replicates from a biologically distinct serum sample. H&E hematoxylin and eosin stain, Cy/Flu cyclophosphamide and fludarabine.

Six months after the last dose of CAR T cells and following multiple disease evaluations confirming remission, surveillance bone marrow biopsy showed hypocellularity and the presence of small aggregates of neoplastic cells with positive desmin immunostaining, indicating relapsed RMS. HER2 expression was maintained on the recurrent BM metastatic disease, although at a lower level by IHC (grade 2, intensity score 2; Fig. 7a). The child was re-enrolled on the trial and infused with the same dose of autologous HER2 CAR T cells (1 × 10^8^ cells/m^2^) after lymphodepletion. Bilateral BMAB evaluation and PET-CT done 6 weeks after re-treatment with CAR T cells showed no morphologic (Fig. 7b) or imaging evidence of RMS, respectively, indicating a CR per the study-specific criteria. After achieving the second CR (CR2), the child completed re-induction with two additional cycles of Cy/Flu and CAR T-cell infusion. Four weeks after the BM demonstrated resolution of metastatic disease, the PD-1-blocking antibody pembrolizumab was added, as allowed on the study protocol, with the intent of promoting the CAR T-cell function. The first dose of pembrolizumab (2 mg/kg/dose) was administered 2 weeks following the second CAR T-cell infusion and every 3 weeks thereafter. The serum IL-15 kinetics during re-induction (Fig. 7c) were comparable to that of the first CR (CR1). The cryopreserved first T-cell product was depleted at the end of re-induction; therefore, a new product was manufactured for infusions during consolidation. The HER2 CAR transduction efficiency (Fig. 7d; gating strategy shown in Supplementary Fig. 7), phenotype (Supplementary Fig. 1c), and cytotoxicity (Supplementary Fig. 1d) were comparable between the two products except for the higher proportion of CD4+ T cells in the second product. During consolidation of CR2, the child received five additional CAR T-cell infusions (1 × 10^8^ cells/m^2^) without lymphodepletion. The total duration of CAR T-cell infusions was 12 months from the attainment of CR2 (Fig. 7e). Bilateral BMAB and whole-body PET-CT were performed 6 weeks after each CAR T-cell infusion during the treatment period and per clinical guidelines thereafter, with no evidence of disease recurrence.

During re-induction, the child developed fever within 12 h of the first and second CAR T-cell infusions, and the grade 1 CRS resolved within 72 h of onset with supportive care. Analysis of serum cytokines showed peripheral blood GM-CSF and IFN-γ levels peak at 4 h after the CAR T-cell infusions given with Cy/Flu, whereas lymphodepletion did not influence the post-infusion TNFα levels (Fig. 7f). In comparison to the induction and consolidation phases of CR1 wherein only CAR T cells were infused, the serum IL-6 and IL-4 levels were substantially higher when the CAR T cells were combined with pembrolizumab during CR2 (Fig. 7g). HER2 CAR T cells expanded in the peripheral blood when infused after Cy/Flu during induction, were better sustained during the consolidation of CR2, and continued to be detected at low levels during off-therapy follow-up (Fig. 7h). The BMAB samples obtained 6 weeks after CAR T-cell infusions showed the presence of the HER2 CAR transgene, but the levels did not directly correlate with that detected in the time-matched peripheral blood (Fig. 7i). The child did not develop a detectable human anti-mouse antibody response despite multiple CAR T-cell infusions. The CR2 is ongoing at the time of this report, 20 months after stopping CAR T-cell infusions.

## Discussion

We report on a child with metastatic RMS who achieved a durable remission after experimental treatment using CAR T cells. Children with high-risk RMS have very poor outcomes^2,12,13^. This child had high-risk RMS at diagnosis based on the unfavorable extremity location of the primary tumor, alveolar-pattern histology, and extensive BM involvement^22^. Treatment with intensive first and second line COG regimens for high-risk RMS could not achieve remission of metastatic disease in this child as evidenced by the detection of neoplastic cells on multiple BM biopsies. This course is consistent with data from previous clinical trials, which showed that intensification of therapy in patients with high-risk RMS failed to improve outcomes^2,22,23^. In children with high-risk disease and CR to upfront therapy, early results from the COG study ARST0431 showed 3-year event-free survival of 20% for those with two or more Oberlin risk factors^12^. In a large cohort of children with metastatic RMS (*n* = 788) treated on nine American and European co-operative group studies, the presence of RMS cells in the BM was shown to be an independent poor prognostic factor^2,22^. The poor prognostic significance of BM metastatic disease in pediatric RMS at diagnosis (*n* = 27) was confirmed in a recent single-institution retrospective study, which found a median survival of 1.5 years from diagnosis in these children^24^. However, 4.3 years after initiating infusions of HER2 CAR T cells, this child with two Oberlin risk factors and chemotherapy-refractory metastatic RMS in the bone marrow remains in remission.

In our preceding dose-escalation study of HER2 CAR T cells^3^, a major limitation identified was the poor expansion and persistence after adoptive transfer. Therefore, we used a combination of Cy/Flu for creating a favorable homeostatic space prior to administering autologous HER2 CAR T cells to this child with refractory disease. This led to an increase in the serum levels of the homeostatic cytokine IL-15 after Cy/Flu and improved expansion of the infused HER2 CAR T cells. The child developed fever and a rise in serum inflammatory markers after each HER2 CAR T-cell infusion during induction, consistent with mild CRS^15^. However, we did not observe any on-target, off-tumor toxicities, and there were no adverse events indicative of lung injury or cardiotoxicity, providing yet another layer of safety data for targeting HER2 with the FRP5-derived CAR T cells^3,25^.

In B cell malignancies, a single dose of CD19-targeted CAR T cells infused after Cy/Flu can show marked expansion, and these cells can subsequently persist at lower frequencies which may be maintained, in part, by sustained exposure to normal CD19-positive B cells^26–28^. These kinetics were predictive of durable remission in CD19-CAR T-cell treated patients^29^. Although BM could represent a favorable disease site due to its accessibility to the circulating CAR T cells, attaining the optimal kinetics needed for disease clearance and durable response is a greater challenge in solid tumors. We initially used Cy/Flu lymphodepletion to facilitate the expansion of HER2 CAR T cells. Then, to consolidate the remission, we used repeated infusions of HER2 CAR T cells alone to sustain their persistence over time. Although this child had a lasting response using our induction and consolidation strategy, recurrent disease was observed 6 months after stopping HER2 CAR T-cell infusions. Tumor recurrence following treatment with CAR T cells targeting a single antigen could result from the emergence of antigen loss variants, and multivalent CAR T cells are being explored as a potential approach to decrease treatment failures^30–35^. Interestingly, the HER2 expression was lower at recurrence, but it remained targetable with CAR T cells in this child and resulted in a CR2 which is currently ongoing.

CAR T cells have direct cytotoxic effects on tumor cells through engagement of the target antigen. Therefore, the disease response achieved here despite the antigenic heterogeneity of the tumor may reflect the dynamic state of HER2 expression and/or the engagement of the host immune response. We examined whether the CAR T-cell therapy altered the adaptive T-cell responses in this child and observed considerable remodeling of the TCRβ repertoire with immunodominant clones during the course of induction. This was accompanied by perturbations of CDR3 loop length distribution and altered TCRβV and TCRβJ gene utilization, suggesting expansion of previously undetected T-cell clonotypes. In a patient with chronic lymphocytic leukemia responding to CTL019 therapy, peripheral expansion of a single T-cell clone with disruption of the *TET2*-gene due to CD19-CAR transgene integration (and hypomorphic mutation in the second allele) was thought to be the predominant mediator of the observed antitumor response^36^. In our patient with RMS, while some pre-existing T-cell clones were sustained through multiple infusions during induction and increased in frequency to >1% of the homeostatic space, we observed bursts of unique TCRβ productive rearrangements occupying a substantial percentage of the clonal space after subsequent infusions of the same CAR T-cell product. Specifically, eight immunodominant clones identified post infusion were not detected in the CAR T-cell product or the pre-infusion peripheral blood, which may indicate an adaptive immune response^37^. Theoretically, lymphodepletion with Cy/Flu could have contributed to the changes in TCRβ rearrangements noted between infusions^38^. However, a recent study of adult patients with hematological or solid malignancies found that the age-specific pre-treatment TCR repertoire is restored after recovery from lymphodepleting chemotherapy^39^. In this child, the distribution of productive CDR3 rearrangements after CAR T-cell infusions given with and without Cy/Flu was distinct in comparison to the pre-infusion peripheral blood obtained 4 weeks after prior cytotoxic chemotherapy only. Given the lack of primary tumor cells and reliable technology to determine the TCR-specificity of the immunodominant clones identified after HER2 CAR T-cell infusion in this patient, our observations currently remain descriptive but warrant further studies, ideally in a larger cohort of responding and non-responding patients.

Further evaluation of the humoral immune response based on the trend in serum IgG level demonstrated an elevated autoantibody response after HER2 CAR T-cell infusions compared to their reactivity in the pre-infusion sample obtained after prior chemotherapy alone. This finding suggests HER2 CAR T-cell-augmented endogenous immune reactivity against various molecules, including those implicated in tumor invasion and metastasis (e.g., FUT8 and USP2)^20,40^. Interestingly, a spike in the serum IgM level was detected at the time of relapse, which was accompanied by a concomitant waning of the autoantibody responses that were present during remission. These changes correlated with the distinct serum IgG autoantibody profile identified during relapse using the proteomic analysis. Taken together, these observations may indicate a unique antigenic profile of the relapsed tumor^11,41^. As antitumor responses are largely mediated by T cells, the autoantibody responses to intracellular proteins such as FUT8, USP2, and GSK3A may represent an ongoing multi-faceted immune response against the tumor, although their functional significance remains uncertain^42^.

The inflammatory milieu resulting from repeated CAR T-cell infusions may have contributed to the upregulation of the immune-modulatory receptors, PD-1 and LAG3, on CAR-expressing CD8+ cytotoxic T cells. The cell-surface upregulation of these receptors likely indicates an activated state of CAR T cells at the time of peak expansion, 7 days after each infusion during the induction phase. PD-1 blockade was reported to reinvigorate CD19-CAR T cells and induce clinical response in an adult patient with B cell lymphoma^43^. In sarcomas, despite pre-clinical studies suggesting a potential role for checkpoint inhibitor therapy, early clinical trials using pembrolizumab or nivolumab as single agents have shown very limited efficacy^44,45^. There are no reports of pediatric sarcoma with CR to anti-PD-1 therapy alone, and pre-clinical studies on expression of PD-L1 in pediatric RMS are limited^46^. In view of the upregulation of PD-1 on CD8+ CAR+ T cells following repeat infusions during CR1, we reasoned that incorporating pembrolizumab with CAR T cells could improve CAR T-cell function and lead to clinical benefit in our patient. During the combinatorial treatment, we observed higher serum levels of Th2 cytokines IL-6 and IL-4, the source and implications of which need to be evaluated in additional patients. As the HER2 CAR T-cell product infused during the consolidation of CR2 was different from that of CR1, it is unclear whether PD-1 blockade had a role in improved sustenance of CAR T cells in the peripheral blood. Now, 20 months after stopping CAR T-cell infusions, this child remains in disease remission.

In conclusion, we report on attaining a durable disease remission in a child with refractory metastatic RMS by safely enhancing the expansion and persistence of autologous HER2-specific CAR T cells. In-depth analysis of the described infusion regimen in a larger cohort of patients with RMS is needed to further evaluate the antitumor efficacy of HER2-specific CAR T cells and the potential contribution of an endogenous immune response against non-targeted proteins in achieving tumor control.

## Methods

### Study design and evaluations

This phase I, open-label, dose-escalation trial (ClinicalTrials.gov identifier: NCT00902044) for advanced sarcoma is designed to primarily assess the safety of one intravenous injection of autologous HER2-specific CAR T cells administered after lymphodepleting chemotherapy. Assessment of in vivo expansion, persistence, and antitumor activity of infused CAR T cells were secondary objectives of the study. Patients who tolerated the initial infusion well and had no clinical decline were eligible to receive additional HER2 CAR T-cell infusions 6–12 weeks apart. The clinical trial protocol is approved by Baylor College of Medicine’s Institutional Review Board (IRB), the recombinant DNA advisory committee of the National Institutes of Health, and the US Food and Drug Administration (FDA). Patients with refractory or recurrent HER2-positive sarcoma are eligible. Concurrent treatment with antineoplastic agents is not allowed 4 weeks before and 6 weeks after the CAR T-cell infusions except for PD-1 or PDL-1 inhibitors. The interim analysis of the phase I study is conducted and reported to the IRB and FDA on an ongoing basis in accordance with the pre-defined study criteria and the regulatory guidelines. The Data Safety Monitoring Committee of the Dan L. Duncan Comprehensive Cancer Center at Baylor College of Medicine is informed of this single patient report from an ongoing clinical trial. This publication describes an exceptional antitumor response observed in one patient following the CARE guidelines for transparency and accuracy of health research reporting and is in line with the study consent obtained at the time of enrollment. The parent(s) of this child provided a written informed consent for participation and reporting of this research study.

HER2 CAR T cells were manufactured from ~60 mL autologous peripheral blood using a gamma-retrovirus delivery system as previously described^3^. The CAR T cells were evaluated for sterility, HLA identity, immunophenotype, and HER2-specific cytotoxicity (4-h chromium-51 release assay) prior to cryopreservation. Lymphodepletion consisted of Cy (30 mg/kg/day) for 2 days followed by Flu (25 mg/m^2^/day) for 5 days (Cy/Flu)^6^. The prescribed HER2 CAR T-cell dose was 1 × 10^8^ cells/m^2^, the highest dose previously tested without lymphodepletion^3,25^. The T-cell dose was based on the total number of cells infused. Study-related adverse events were collected and analyzed as per Common Terminology Criteria for Adverse Events with the exception of CRS, which was graded according to Lee et al.^15^. Disease evaluation was performed with whole-body PET-CT and bilateral BMAB prior to study entry and at 6 weeks after each T-cell infusion to assess the treatment response. Histologic BMAB response was tested and interpreted by an independent clinical pathologist per standard of care. Radiographic responses were evaluated using Response Evaluation Criteria in Solid Tumors (RECIST)^47^. Soluble serum cytokines were quantified using the Luminex^®^ Multiplex Assay (Cat# HCYTOMAG-60K, Lot# 2977010, Millipore, Austin, TX)^3^.

### Immunohistochemistry

HER2 expression was evaluated using immunohistochemistry (mouse monoclonal to ErbB2 [CB11] ab8054; Abcam Inc, Cambridge, MA)^3,48^. HER2 expression was graded for percent positive tumor cells (grade 0, no staining; grade 1, 1–25%; grade 2, 26–50%; grade 3, 51–75%; grade 4, 76–100%) and intensity of staining (0, 1+, 2+, or 3+) as scored by an independent pathologist and compared to HER2-positive breast cancer density gradient tissue microarrays as positive controls. To meet study entry eligibility, tumors were required to have at least grade 1 (1–25% positive) and intensity score 1+ for HER2 staining. Desmin and myogenin immunohistochemistry were performed per standard of care in a CLIA-certified clinical laboratory and were interpreted by an independent pathologist.

### Quantitative polymerase chain reaction

We used qPCR amplification to detect HER2 CAR T cells in patient samples^3^. Briefly, DNA was extracted (QIAamp DNA Blood Mini Kit, Quiagen, Hilden, Germany) from peripheral blood or bone marrow; using FRP5-specific primers (forward primer: 5′-CCACGGTCACCGTTTCCT-3′ 18 bp, reverse primer: 5′-GGGTCAGCTGGATGTCAGAAC-3′ 21 bp, probe (on reverse strand): 5′-FAM-CCGCCACCAGAACCG-NFQ-3′ 15 bp) and TaqMan probe (Applied Biosystems, Waltham, MA). qPCR was performed in triplicates using the ABI 7900HT Sequence Detection System (Applied Biosystems). The baseline range was set at cycles 6–15, with the threshold 10 standard deviations above the baseline fluorescence. Serial dilution of DNA plasmids encoding each cassette was used to generate DNA standards.

### Flow cytometry

A BD Accuri^TM^ C6 (BD Accuri C6 Software, Becton Dickinson, San Jose, CA) or a Gallios flow cytometer (Kaluza Analysis Software, Beckman Coulter, Indianapolis, IN) and FlowJo software v10 (FlowJo LLC, Ashland, OR) were used for flow cytometric analysis of peripheral blood and bone marrow samples. The following monoclonal antibodies were used: mouse anti-human CD3 FITC (stock solution, 5 µL/100 µL test at 1:20 dilution, clone: SK7, Cat# 349201, Lot# 7221481, Becton Dickinson Biosciences, San Jose, CA), mouse anti-human CD8 Pacific Blue (stock solution, 5 µL/100 µL test at 1:20 dilution, clone: B9.11, Cat# A82791, Lot# 38, Beckman Coulter, Indianapolis, IN), mouse anti-human CD279 (PD-1) APC (stock solution, 5 µL/100 µL test at 1:20 dilution, clone: MIH4, Cat# 558694, Lot# 8012764, Becton Dickinson Biosciences, San Jose, CA), mouse anti-human CD223 (LAG3) FITC (stock solution, 5 µL/100 µL test at 1:20 dilution, clone: 3DS223H, Cat# 11-2239-42, Lot# 4337363, Thermo Fisher Scientific, Waltham, MA). HER2 CAR expression in the T-cell product was detected using Alexa Fluor^®^ 647 AffiniPure Goat Anti-Mouse IgG, F(ab′)2 fragment specific (reconstituted, 1:100 to 1:800 dilution, 0.5–1 µL/100 µL test, polyclonal, Cat# 115-605-006, Lot# 122475, The Jackson Laboratory, Bar Harbor, ME), and in the peripheral blood T cells using a recombinant human ErbB2/HER2 Fc chimeric protein (100 µg/mL concentration, 5 µL/100 µL test at 1:20 dilution, Cat# 1129-ER, Lot# FXR0917071, R&D Systems, Minneapolis, MN) followed by a goat anti-human IgG Fc secondary antibody PE (pre-titrated, 1 uL/100 µL test at 1:100 dilution, polyclonal, Cat# 12-4998-82, Lot# 2070588, Thermo Fisher Scientific, Waltham, MA). Negative controls included isotype antibodies and where appropriate, non-transduced T cells stained with the test antibodies. Gating strategy for all flow cytometry experiments is included in Supplementary Information.

### TCRβ CDR3 sequencing

Genomic DNA was extracted using the DNeasy Blood & Tissue Kit (Qiagen, Germantown, MD) per manufacturer’s instructions. The immunosequencing of the CDR3 regions of human TCRβ chains was performed using the ImmunoSEQ^TM^ Assay (Adaptive Biotechnologies, Seattle, WA)^49^. Briefly, genomic DNA was amplified in an unbiased multiplex PCR assay with a mixture of primers (Adaptive Biotechnologies, Seattle, WA)^49^ targeting the rearranged variable and joining segments and a high-throughput DNA sequencing was performed. The sequences were quantitated and analyzed to identify frequency of productive TCRβ CDR3 rearrangements in samples using the ImmunoSEQ Analyzer 3.0 software provided by Adaptive Biotechnologies. Sample clonality and Morisita index were calculated using the ImmunoSEQ Analyzer 3.0 software. For other graphical presentations, data were exported as a tab-separated values file and graphs were plotted on Microsoft Excel. All associated Venn plots were created from the exported data using the webtool found at http://bioinformatics.psb.ugent.be/webtools/Venn/.

### ProtoArray^TM^ human protein microarray

High-throughput serological analysis for autoantibody generation was performed on patient serum samples using the ProtoArray^TM^ Human Protein Microarray (Thermo Fisher Scientific, Rockford, IL)^50^. The reactivity of antibodies present in the patient’s serum to 9480 unique protein probes was investigated by the manufacturer’s antibody profiling services. Briefly, the patient’s serum at pre infusion and 6 weeks post infusions #1, #2, #3, #7 (remission) and relapse at day 546 was profiled at 1:500 dilution and bound antibodies were developed with AF647 goat anti-human IgG. Negative controls were incubated with buffer containing no serum prior to incubation with the Alexa Fluor™647-anti-human IgG detection reagent. The background values for the assay ranged from 39 to 61 RFU. Pixel intensities for each spot were determined using GenePix 7 software and analyzed using Thermo Fisher Scientific’s proprietary ProtoArray™ Prospector software.

In conducting the analyses comparing selected individual serum samples, the data from one sample were filtered to remove proteins that were bound by the anti-human IgG detection reagent alone and compared in pairwise fashion to a second sample. Proteins were defined as eliciting an increased signal from serum autoantibody binding if they met the following criteria: (1) the Signal Used value, or background-subtracted signal, on the array probed with one sample under comparison was at least 5000 RFU; (2) the ratio of the Signal Used value on the array probed with one sample under comparison to the Signal Used value on the other array under comparison was >2; (2) the *z*-score, or normalized signal intensity, was <1.0 on the negative control array. The *z*-score indicates the number of standard deviations above or below the median signal value for all human protein features on the array; (4) the Z-factor, or signal-to-noise ratio, was >0.5 on the corresponding array, indicating a signal greater than twofold above the noise; (5) the coefficient of variation was <50% for the adjacent replicate spots on the corresponding array; and (6) a CI *P* value < 0.05 as calculated by the ProtoArray^®^ Prospector software. The CI *P* value assigns a probability that an observed signal is derived from the distribution of signals arising from a set of defined negative controls. Typically, a CI *P* value < 0.05 correlates with a visually confirmable signal on the array. Cytoscape maps depicting nodes of genes and informative functional terms were visualized using the WebGIVI tool (http://raven.anr.udel.edu/webgivi/)^51^.

### Indirect ELISA

The serum IgG and IgM levels at various time points over the course of treatment (pre-infusion, 6 weeks post each infusion during CR1 and at relapse) were determined using IgG (total) Human uncoated ELISA kit (Cat# 88-50550-22, Lot# 175941117) and IgM Human uncoated ELISA kit (Cat# 88-50620-22, Lot# 1666010115), respectively, as per manufacturer’s instructions (Invitrogen, Carlsbad, CA). Indirect ELISA was performed to validate the reactivity of patient serum to rFUT8, rUSP2, rRAB7B, and rGSK3A. Briefly, 96-well ELISA plates were coated with recombinant proteins (1 µg/ml; 100 µl/well; Abcam, Cambridge, MA) in carbonate buffer. After blocking with 2.5% Milk-PBS-T20, the patient’s plasma collected at pre infusion and post infusion time points was incubated for an hour at 1:125, 1:250, 1:500, and 1:1000 dilutions. Goat anti-human IgG (γ-chain specific) conjugated to HRP (1:2500 dilution; Cat# A8419-2ML, Lot# 077M4873V, Sigma-Aldrich, St. Louis, MO) was used as secondary antibody and the assay was developed with TMB substrate (BioLegend, San Diego, CA). The reaction was stopped after 15 min with 2.5 M sulfuric acid and read at 450 nm using an Infinite^®^ F50 microplate reader (Tecan, Switzerland).

### Statistical analysis and reproducibility

Data were generated using biologically distinct samples when possible, employing technical replicates in each experiment as indicated. All experimental results were appropriately repeated for validation except in the scenarios where the patient sample was limited. Specifically, flow cytometry analysis of the post-infusion PBMC was optimized and repeated using donor PBMC with decreasing concentrations of CAR T cells to ensure reproducibility prior to testing of patient sample(s). Disease evaluation with histopathological examination of the bone marrow and whole-body PET-CT was done as part of patient care following standard clinical guidelines. GraphPad Prism 8.0 or Microsoft Excel 2013 was used for data analysis and graphical presentation. All data were summarized using descriptive statistics as mean ± SD.

### Reporting summary

Further information on research design is available in the Nature Research Reporting Summary linked to this article.

## Supplementary information

Supplementary Information

Reporting Summary

## Data Availability

The source data underlying Figs. 1f, 2a–e, 3d–g, 4–6, 7c, d, f–i and Supplementary Figs. 1a–d, 2a–e, and 3a, b are provided as a Source Data file. ProtoArray^TM^ data in this publication are deposited in NCBI’s functional genomic data repository Gene Expression Omni3a, bbus (GEO) and are accessible through GEO series accession number GSE152029. Figures 1g and 7e are created by S.N. using BioRender.com (purchased license). For these figures, timeline from initial study enrollment is provided without the dates to protect the research participant’s privacy, and to align with the study consent. All the other data supporting the findings of this study are available within the article, supplementary information, source files, and from the corresponding author upon reasonable request. Source data are provided with this paper.

## Source data

Source Data
