# Supplementary material for: Tumor response and endogenous immune reactivity after administration of HER2 CAR T cells in a child with metastatic rhabdomyosarcoma
Source: Nat Commun. 2020 Jul 15;11:3549. doi: 10.1038/s41467-020-17175-8 (PMC7363864; doi:10.1038/s41467-020-17175-8)
Supplement: Supplementary file 1 — Supplementary Information [file 41467_2020_17175_MOESM1_ESM.pdf]

Supplementary Information

Tumor response and endogenous immune reactivity after administration of HER2 CAR T cells in a child with metastatic rhabdomyosarcoma

Hegde M et al

Supplementary Appendix

Table of Contents

Supplementary Figures

Supplementary Figure 1 page 3
Supplementary Figure 2 page 5
Supplementary Figure 3 page 6
Supplementary Figure 4 page 7
Supplementary Figure 5 page 8
Supplementary Figure 6 page 11
Supplementary Figure 7 page 12

Supplementary Tables

Supplementary Table 1 page 13
Supplementary Table 2 page 14
Supplementary Table 3 page 15
Supplementary Table 4A page 16
Supplementary Table 4B page 17
Supplementary Table 4C page 19
Supplementary Table 5A page 20
Supplementary Table 5B page 21
Supplementary Table 6 page 22
Supplementary Table 7 page 24

Supplementary References page 25

Supplementary Figures

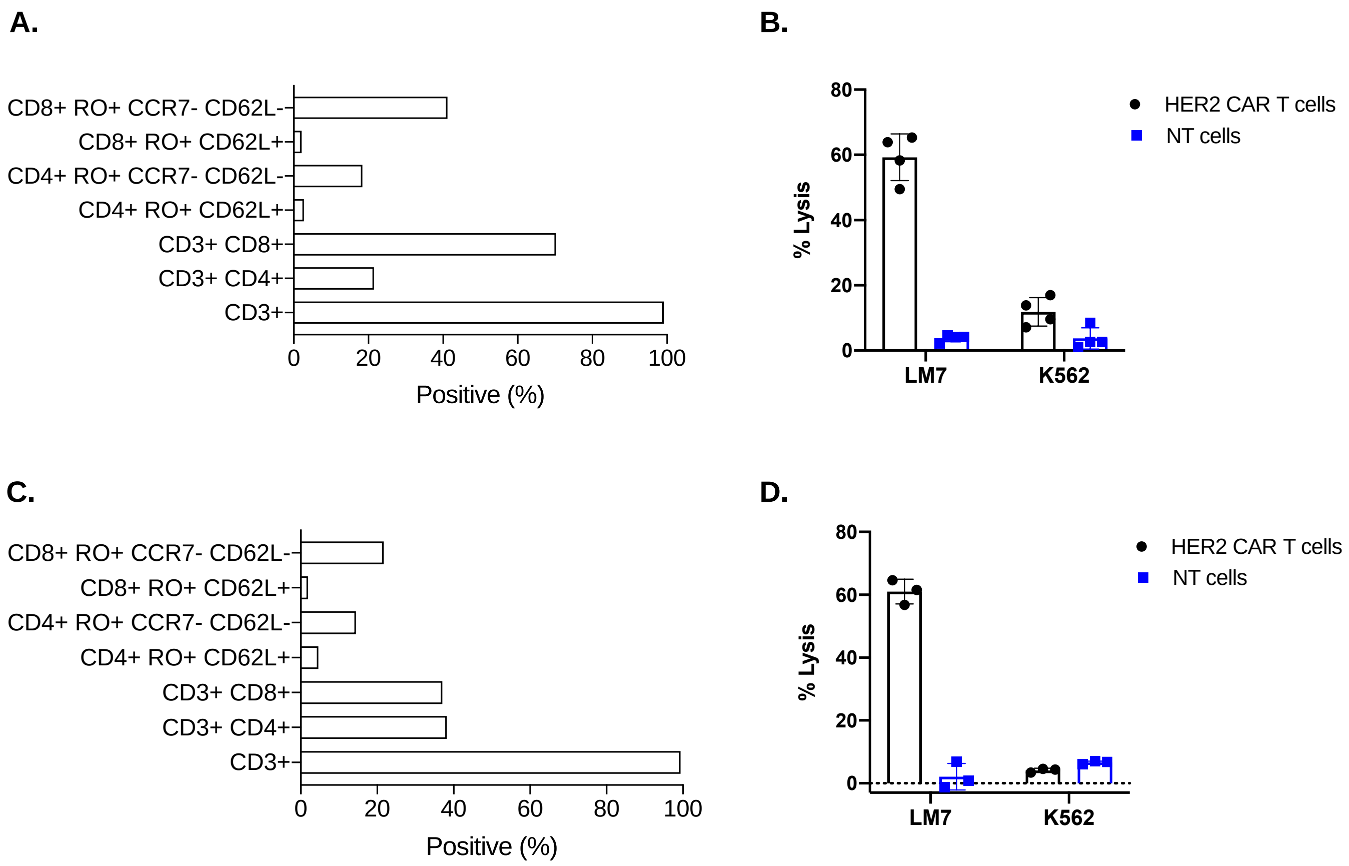

Supplementary Figure 1: Characterization of the autologous HER2 CAR T-cell products. (A) Component analysis and phenotype of the first cellular product. The cells were predominantly CD8+ T-cell subsets with effector memory phenotype. (B) Cytolytic activity of the HER2 CAR T cells (first product) against HER2-positive (LM7) and HER2-negative (K562) tumor targets. Non-transduced (NT) T cells were used as controls in 4-hour chromium-51 release assay; n=4 technical replicates, data are presented as mean values +/- SD. (C) Component analysis and phenotype of the second HER2 CAR T-cell product. The second product contained a higher proportion of CD4+ T cells with predominantly effector memory phenotype. (D) Cytolytic activity of the HER2-CAR T cells (second product) against HER2-positive (LM7) and HER2-negative (K562) tumor targets; n=3 technical replicates, data are

presented as mean values +/- SD. Manufactured CAR T-cell product is tested for cytolytic activity using two HER2-positive and two HER2-negative tumor cell lines at 4 effector to target ratios (5:1, 10:1, 20:1, 40:1). Representative graphs in panel (B) and (D) depict the cytolytic activity against one HER2-positive and one HER2-negative tumor target at 20:1 effector to target ratio. CAR T-cell product component analysis is done using a standard flow cytometry panel developed in the Good Manufacturing Practice (GMP) Facility at Texas Children's Hospital.

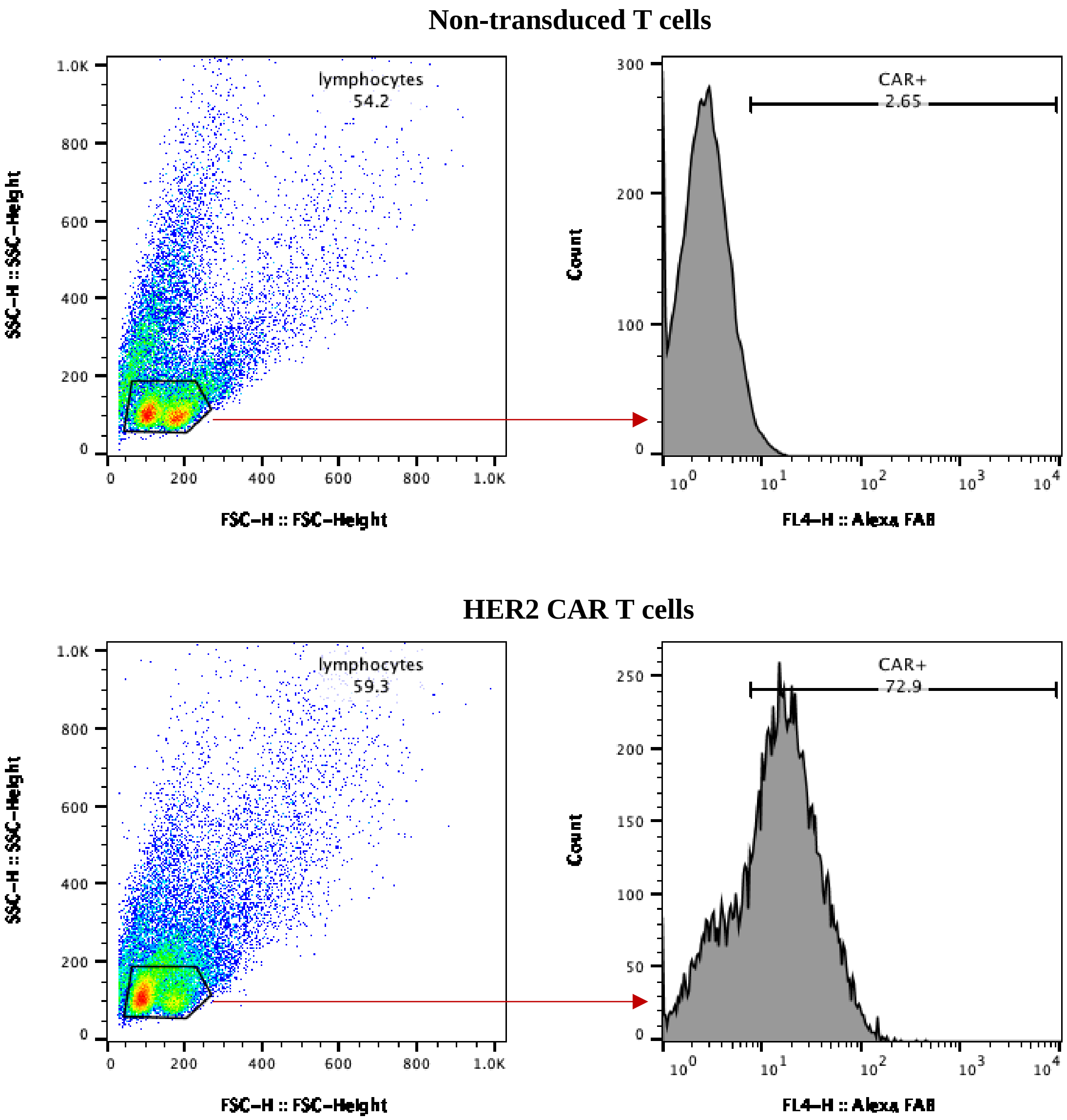

Supplementary Figure 2: Gating strategy for HER2 CAR transduction on T cells shown in Figure 1F. Flow cytometry was performed on the patient's T cells at approximately one week after retroviral CAR transduction and repeated prior to product freezing. Histograms shown are representative data. Lower panel demonstrates the gating strategy for evaluating the HER2 CAR expression on patient's T cells in comparison to non-transduced T cells which is shown in the upper panel.

Peripheral Blood Mononuclear Cells

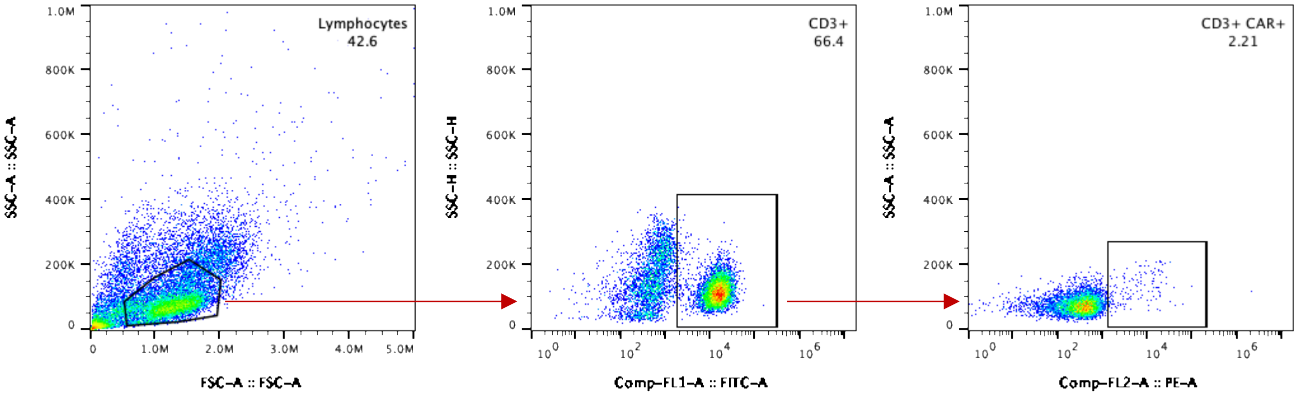

Supplementary Figure 3: Gating strategy for detection of HER2 CAR T cells in the peripheral blood using flow cytometry shown in Figures 3D and 3E. Flow cytometry was performed on patient's peripheral blood mononuclear cells (PBMC) at 7 days after the CAR T-cell infusion. HER2 CAR expressing T cells were detected using a recombinant ErbB2/HER2 Fc chimeric protein followed by a goat anti-human IgG Fc secondary antibody as described in methods section of the manuscript. The assessment of the peripheral blood was repeated for each CAR T cell infusion given after lymphodepletion. Representative dot plots are shown here to demonstrate the gating strategy used.

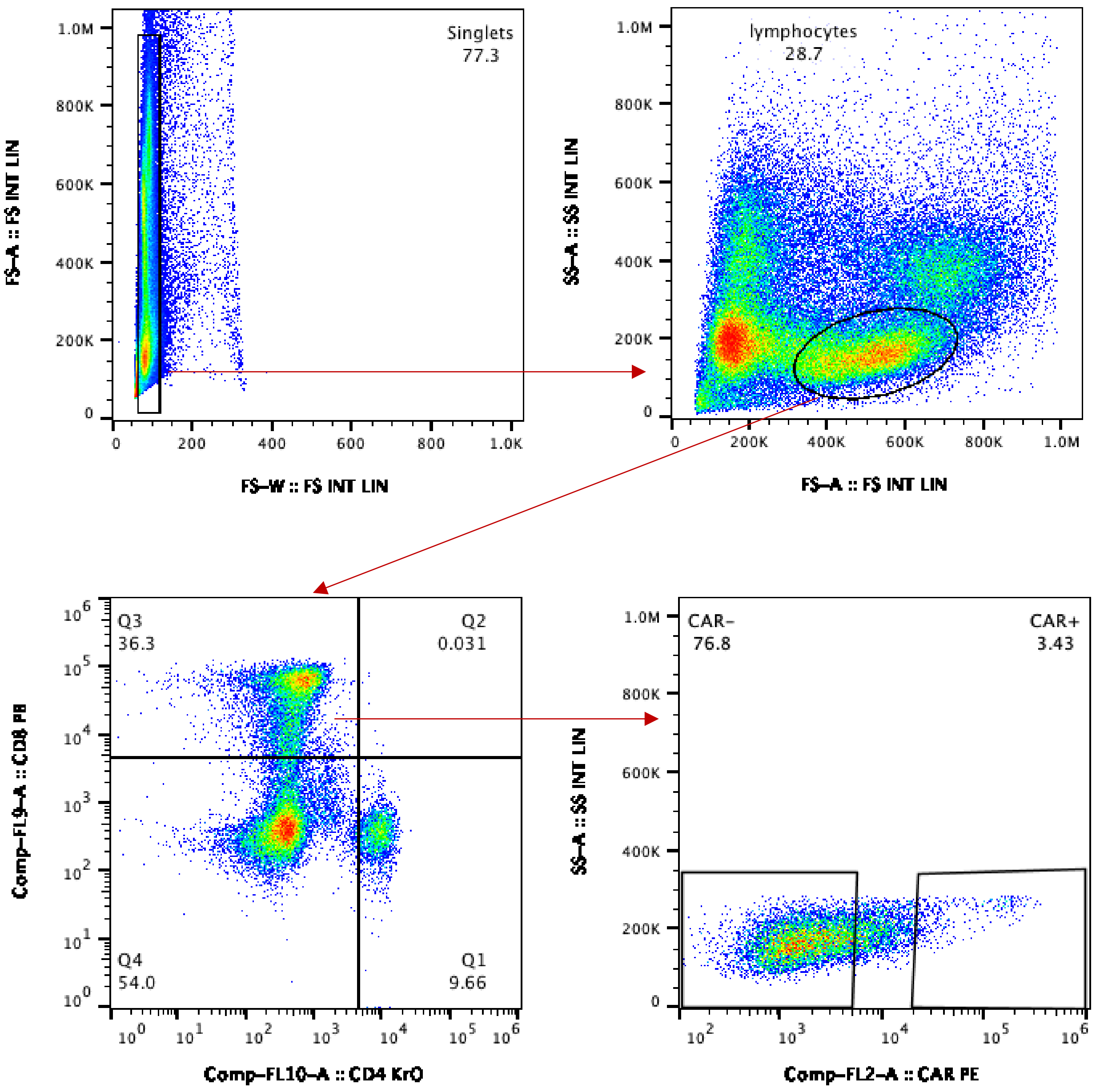

Supplementary Figure 4: Gating strategy for analysis of PD1 and LAG3 surface expression on peripheral blood CD8+ T cells shown in Figures 3F and 3G. Flow cytometry was performed on peripheral blood at 7 days after the HER2 CAR T-cell infusion to evaluate the surface expression of PD1 and LAG3 on CD8+ T cells as described in manuscript methods. Representative dot plots shown here depict the gating strategy used for analysis.

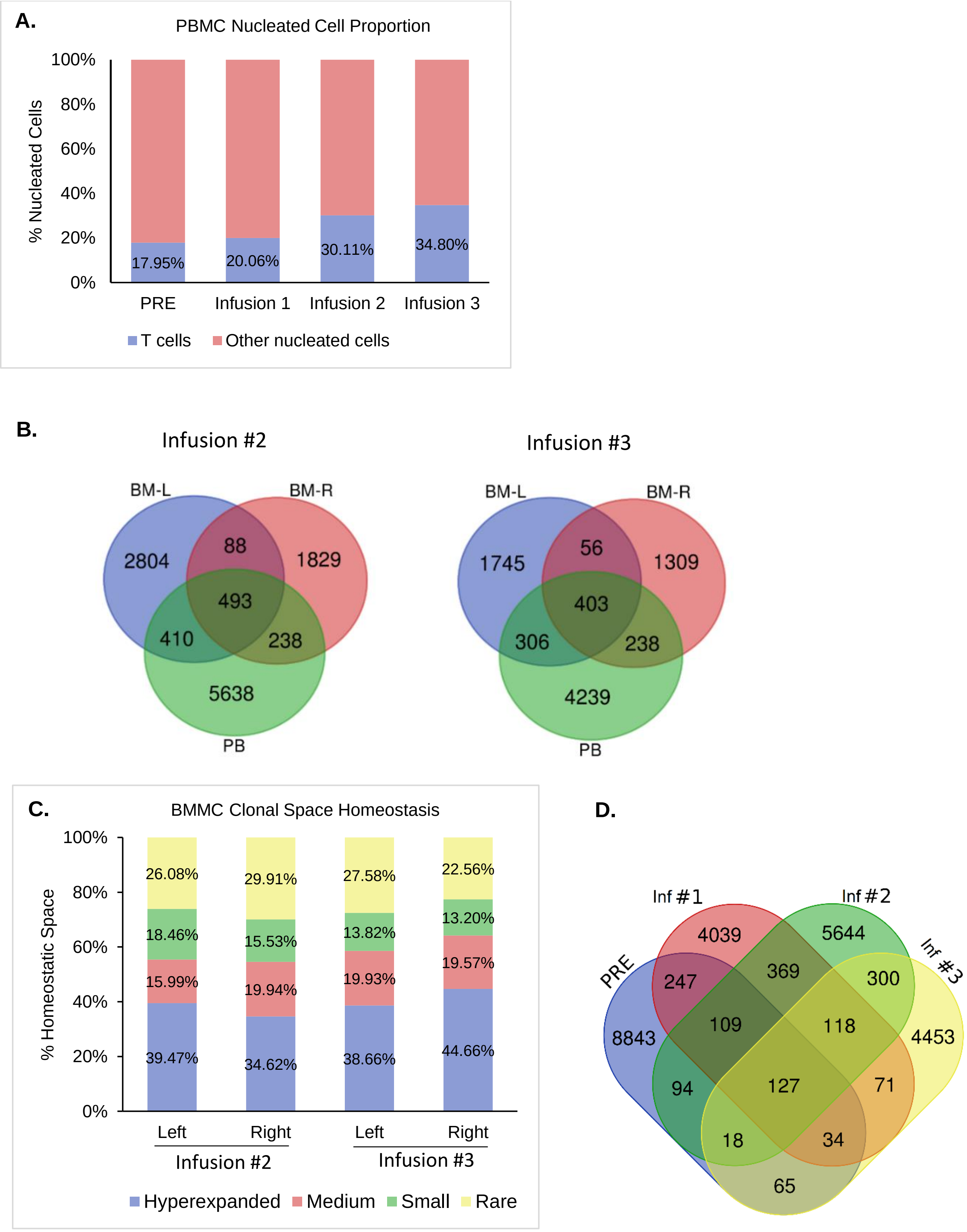
A.
PBMC Nucleated Cell Proportion
% Nucleated Cells
100%
80%
60%
40%
20%
0%
17.95%
20.06%
30.11%
34.80%
PRE
Infusion 1
Infusion 2
Infusion 3
T cells
Other nucleated cells
B.
Infusion #2
BM-L
BM-R
2804
88
1829
493
410
238
5638
PB
Infusion #3
BM-L
BM-R
1745
56
1309
403
306
238
4239
PB
C.
BMMC Clonal Space Homeostasis
% Homeostatic Space
100%
80%
60%
40%
20%
0%
26.08%
18.46%
15.99%
39.47%
29.91%
15.53%
19.94%
34.62%
27.58%
13.82%
19.93%
38.66%
22.56%
13.20%
19.57%
44.66%
Left
Right
Infusion #2
Left
Right
Infusion #3
Hyperexpanded
Medium
Small
Rare
D.
Inf #1
Inf #2
PRE
Inf #3
4039
5644
247
369
300
8843
109
118
4453
94
127
71
18
34
65

E.

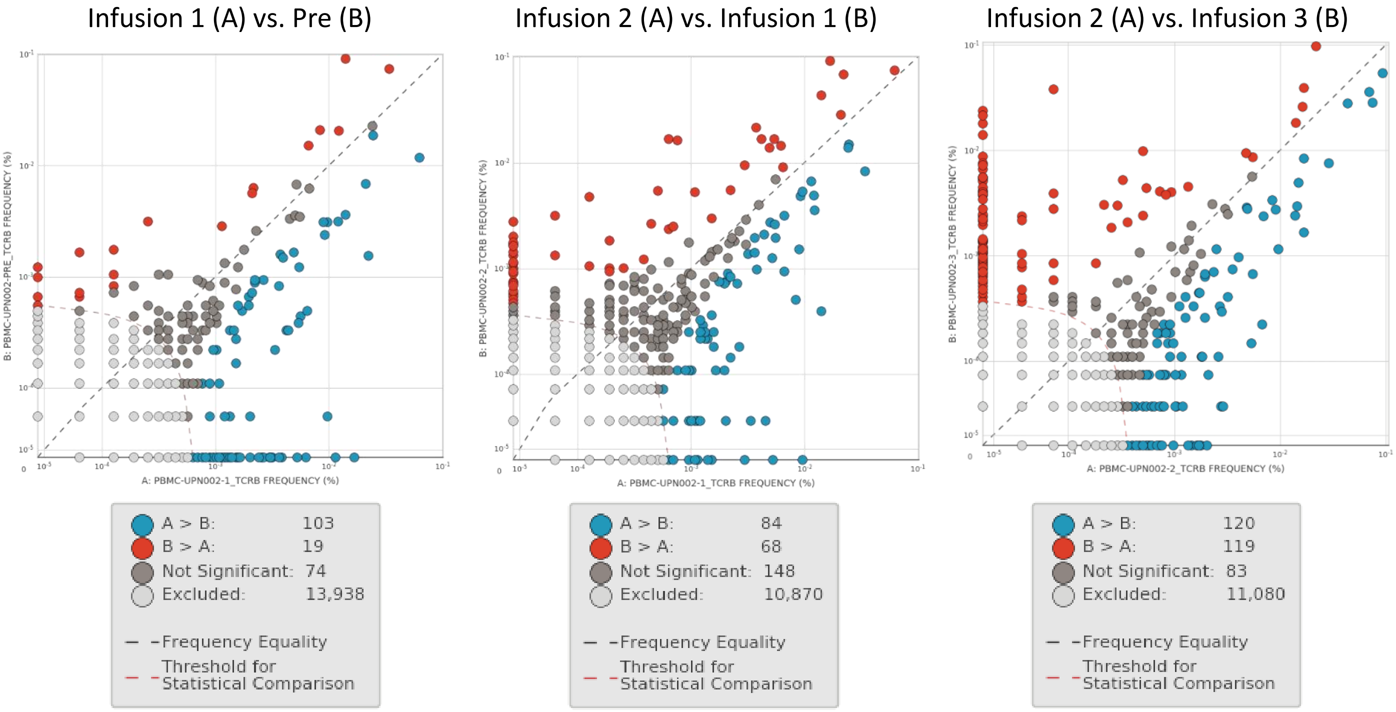

Supplementary Figure 5: Longitudinal tracking of T-cell clones in the peripheral blood and bone marrow during TCRβ repertoire remodeling. (A) Longitudinal tracking of the proportion of nucleated cells in the peripheral blood before and after HER2 CAR T-cell infusions. PBMC, peripheral blood mononuclear cells. (B) Venn diagram representing the unique TCRβ CDR3 rearrangements (amino acids) between the bone marrow (BM) metastatic sites and peripheral blood (PB) after the second and third CAR T-cell infusions. L, Left. R, Right. (C) Homeostatic space distribution of T-cell clones from the bone marrow categorized as hyperexpanded/large (>1% frequency of productive rearrangements), medium (0.1-1% frequency), small (more than single event, but less than 0.1% frequency) and rare (single rearrangement events) 6 weeks after the second and third CAR T-cell infusions. (D) Venn diagram of productive CDR3 rearrangements (amino acids) in the peripheral blood before (PRE) and 6 weeks after the first, second and the third CAR T-cell infusions (Inf). (E) Differential abundance based on increasing

or decreasing frequencies of unique TCRβ CDR3 rearrangements (amino acids) between pre-infusion and post-infusion #1, post-infusion #1 and post-infusion #2 as well as post-infusion #2 and post-infusion #3 samples from the peripheral blood obtained during the initial induction period.

A.

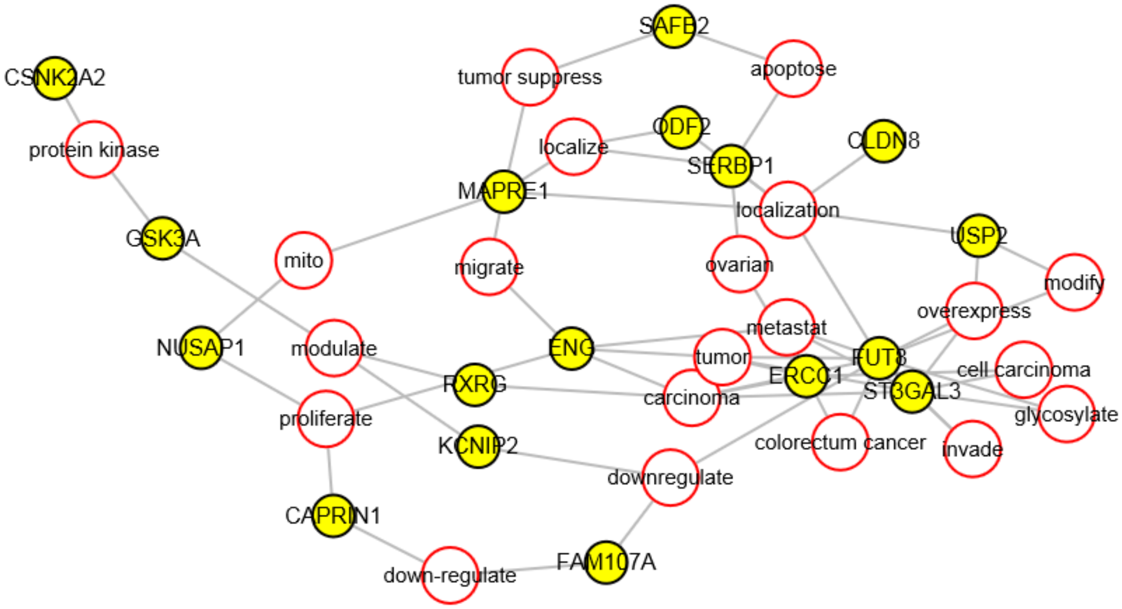

B.

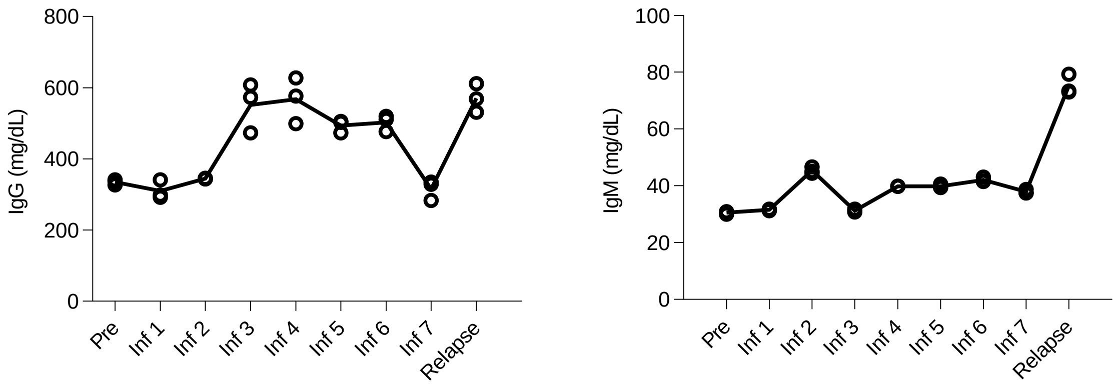

Supplementary Figure 6: Serum antibody responses identified after HER2 CAR T-cell infusions. (A) Cytoscape plot of tumor-related genes analyzed using the WebGIVI tool from the ProtoArray™ Human Protein Microarray results. Yellow shaded circles denote genes and red circles denote functional nodes. (B) Trend in serum IgG and IgM levels determined by indirect ELISA prior to study initiation and during the treatment course (6 weeks after each HER2 CAR T-cell infusion) and at disease recurrence, six months after stopping CAR T-cell infusions. Pre, pre-infusion. Inf, CAR T-cell infusion.

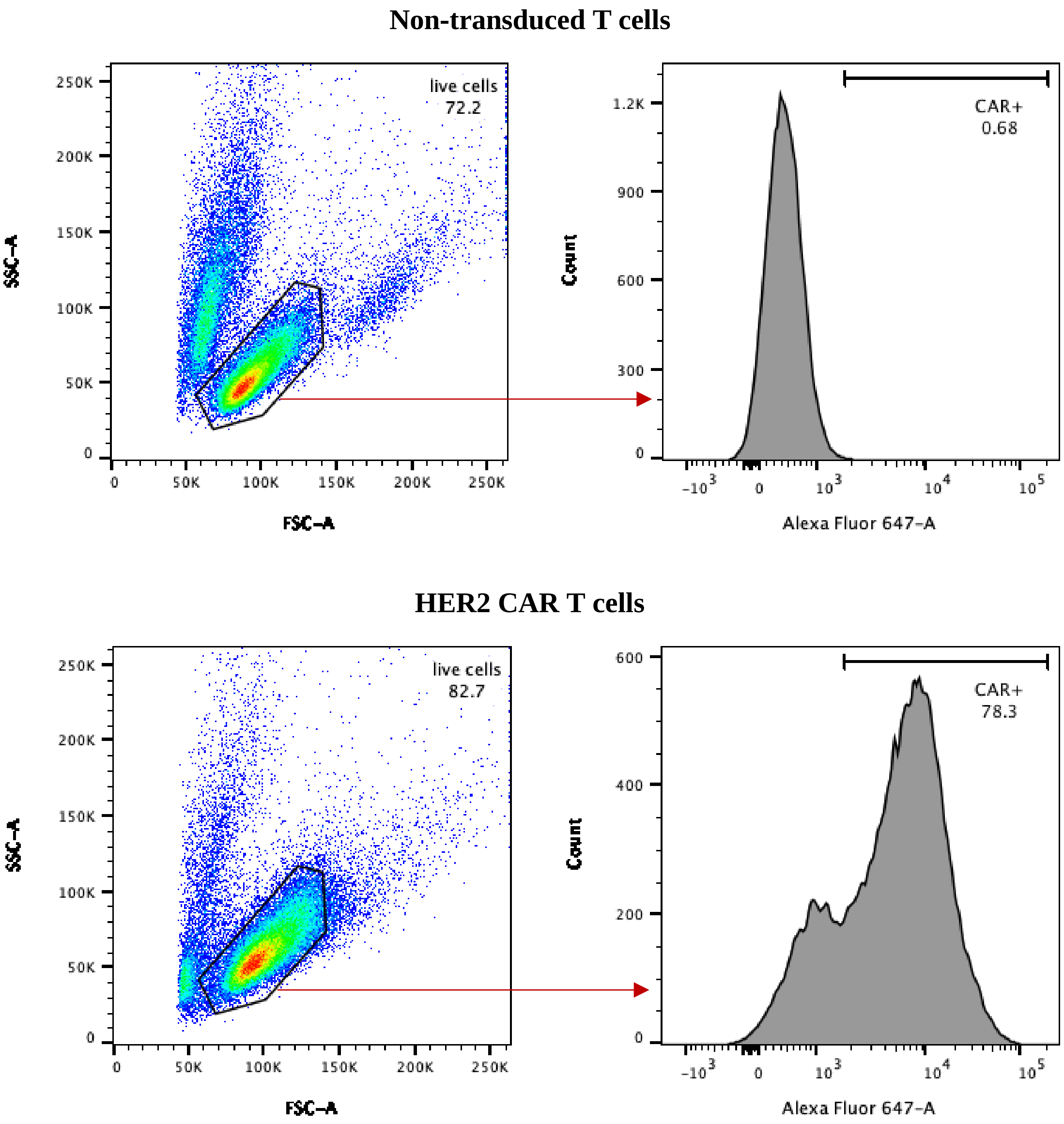

Supplementary Figure 7: Gating strategy for HER2 CAR transduction on T cells shown in Figure 7D. Flow cytometry was performed on the patient's T cells at approximately one week after retroviral CAR transduction and repeated prior to product freezing. Histograms shown are representative data. Lower panel demonstrates the gating strategy for evaluating the HER2 CAR expression on patient's T cells in comparison to non-transduced T cells which is shown in the upper panel.

Supplementary Tables

Supplementary Table 1. Summary of the patient's first- and second-line treatment for high-risk rhabdomyosarcoma given according to the Children's Oncology Group (COG) protocols, ARST0431 and ARST0921, respectively.

Protocol: ARST0431			
Agent	Dose		Cycles (in order of administration)
V	1.5mg/m^2 IV x 1		(IRIN-V) x2
IRIN	50mg/m^2/day IV x 5		Disease Evaluation
I	1800 mg/m^2/day IV x 5		(VDC/IE) x6
E	100 mg/m^2/day x 5		Disease Evaluation
D	37.5 mg/m^2/day x 2		(IRIN-V/IE/VDC) x6 + XRT to primary site
C	1200 mg/m^2 IV x1		Disease Evaluation
A	0.045 mg/kg IV x 1		(VAC/IRIN-VCR) x6
Protocol: ARST0921			
Agent	Dose		Cycles
TEMS	15 mg/m^2 IV x 3 (days 1, 8, 15)		(VINO/C + TEMS) x2
VINO	25 mg/m^2 IV x 2 (days 1, 8)		
C	1200 mg/m^2 IV x 1 (day 1)		

V: Vincristine, IRIN: Irinotecan, I: Ifosfamide, E: Etoposide, D: Doxorubicin, C: Cyclophosphamide, A: Dactinomycin, XRT: Radiotherapy, TEMS: Temsirolimus, VINO: Vinorelbine. MESNA was administered with C and I. PEG-filgrastim was administered with VAC, VD and IE cycles.

Supplementary Table 2. Adverse events reported during the first enrollment and study follow up period.

Adverse Event	Grade 1	Grade 2	Grade 3	Grade 4
Hematological				
Lymphopenia	C-2	C-1	-	I-1, I-2, I-3
Neutropenia	-	-	I-1	I-2, I-3
Leukopenia	-	C-1, C-2, C-4	-	I-1, I-2, I-3
Gastrointestinal				
Nausea	I-3	-	-	-
Vomiting	I-3	-	-	-
Elevated AST	I-3	-	-	-
Elevated alk phos	I-2, C-1, C-2, C-3			
Electrolyte disturbances				
Hyponatremia	I-1, I-2, I-3	-	-	-
Constitutional				
Fever	-	I-1, I-2, I-3	-	-
Chills	I-3	-	-	-
Fatigue	I-3	-	-	-
Anorexia	I-3	-	-	-
Immunological				
CRS	I-1, I-2, I-3	-	-	-

I: induction cycle, C: consolidation cycle, AST: Aspartate aminotransferase, alk phos: alkaline phosphatase, CRS: cytokine release syndrome

Supplementary Table 3. Sample overview of TCRβ CDR3 survey sequencing.

Sample	Total CDR3 Rearrangements	Productive Rearrangements	% Productive Rearrangements	Sample Clonality	Total Nucleated cells	Total T cells	% T cells
HER2 CAR T cell product	72985	59276	0.81	0.024	83424	68328	81.90%
PB PRE	12197	9655	0.79	0.195	91771	16472	17.95%
PB Infusion 1	6640	5204	0.78	0.199	72413	14523	20.06%
PB Infusion 2	8707	6899	0.79	0.332	86056	25914	30.11%
PB Infusion 3	6715	5283	0.79	0.331	70685	24596	34.80%
PB Infusion 4	31626	25957	0.80	0.139	94076	38680	41.11%
PB Infusion 6	23906	20055	0.82	0.118	80113	26462	33.03%
BM Left Infusion 2	4871	3856	0.79	0.262	141593	10422	7.36%
BM Right Infusion 2	3382	2682	0.79	0.229	109514	6310	5.76%
BM Left Infusion 3	3175	2560	0.81	0.226	87510	6880	7.86%
BM Right Infusion 3	2592	2041	0.79	0.248	71239	6353	8.92%
BM Left Infusion 4	5643	4591	0.79	0.146	139626	7602	5.44%
BM Right Infusion 4	7082	5761	0.79	0.180	116178	10554	9.08%

TCR, T-cell receptor. CDR3, complementarity determining region 3. PB, peripheral blood. PRE, pre-infusion. BM, bone marrow.

Supplementary Table 4A. TCRβ CDR3 length use variation in the peripheral blood (PB) prior to study initiation and at 6 weeks after each HER2 CAR T-cell infusion during the induction of first remission.

Amino acid	Nucleotide	PB pre-infusion	PB post-infusion 1	PB post-infusion 2	PB post-infusion 3
7	21	0.011%	0.013%	0.011%	0.011%
8	24	0.011%	0.019%	0.011%	0.000%
9	27	0.061%	0.025%	0.025%	0.041%
10	30	0.322%	0.361%	0.304%	0.201%
11	33	1.853%	1.129%	1.140%	1.088%
12	36	17.739%	11.883%	5.517%	13.599%
13	39	14.593%	16.278%	9.731%	11.424%
14	42	16.330%	21.712%	20.345%	15.067%
15	45	25.291%	21.313%	16.059%	21.466%
16	48	11.664%	16.804%	33.295%	25.120%
17	51	5.410%	5.954%	9.087%	7.770%
18	54	2.969%	1.909%	1.770%	1.453%
19	57	3.046%	1.604%	0.695%	1.702%
20	60	0.427%	0.881%	1.857%	0.972%
21	63	0.178%	0.082%	0.101%	0.063%
22	66	0.061%	0.032%	0.015%	0.004%
23	69	0.022%	0.000%	0.025%	0.011%
24	72	0.011%	0.000%	0.007%	0.007%
25	75	0.000%	0.000%	0.000%	0.000%
26	78	0.000%	0.000%	0.004%	0.000%

Supplementary Table 4B. TCRβV family gene use in the CDR3 region of peripheral blood (PB) T cells before and 6 weeks after the first, second and third HER2 CAR T-cell infusions.

TCRB V Gene	PB pre-infusion	PB post-infusion 1	PB post-infusion 2	PB post-infusion 3
--	0.1054%	0.0444%	0.072%	0.022%
TCRBV01-01	0.0333%	0.0190%	0.065%	0.011%
TCRBV02-01	3.6455%	8.0342%	13.482%	9.401%
TCRBV03-01/03-02	2.7633%	2.5491%	1.350%	1.140%
TCRBV04-01	1.5648%	1.5282%	1.220%	1.520%
TCRBV04-02	0.7657%	0.6341%	0.492%	0.216%
TCRBV05	0.0055%	0.0000%	0.007%	0.004%
TCRBV05-01	3.9785%	3.0691%	2.140%	2.183%
TCRBV05-03	0.1831%	0.0507%	0.047%	0.019%
TCRBV05-04	1.8311%	1.9087%	1.151%	1.114%
TCRBV05-05	2.4359%	3.7032%	2.947%	1.088%
TCRBV05-06	1.8255%	1.9341%	1.068%	1.114%
TCRBV05-07	0.0000%	0.0127%	0.011%	0.004%
TCRBV05-08	0.4217%	0.3044%	0.206%	0.253%
TCRBV06	1.2152%	1.0780%	0.702%	0.566%
TCRBV06-01	1.5148%	1.5916%	3.030%	3.974%
TCRBV06-02	0.1554%	0.1712%	0.062%	0.063%
TCRBV06-02/06-03	0.4051%	0.3614%	0.167%	0.183%
TCRBV06-04	0.5216%	0.1902%	0.337%	0.168%
TCRBV06-05	5.0938%	6.7280%	5.600%	12.266%
TCRBV06-06	2.6912%	1.3063%	0.612%	1.240%
TCRBV06-07	0.0166%	0.0127%	0.004%	0.007%
TCRBV06-08	0.0000%	0.0127%	0.007%	0.007%
TCRBV06-09	0.0055%	0.0000%	0.011%	0.007%
TCRBV07	0.1776%	0.1268%	0.087%	0.652%
TCRBV07-02	0.8268%	0.9956%	0.786%	0.685%
TCRBV07-03	1.0820%	2.2955%	1.658%	0.756%
TCRBV07-04	0.0111%	0.0190%	0.015%	0.015%
TCRBV07-05	0.0166%	0.0063%	0.011%	0.000%
TCRBV07-06	1.0765%	0.4883%	0.460%	0.358%
TCRBV07-07	0.0721%	0.0888%	0.051%	0.045%
TCRBV07-08	1.0043%	0.8180%	0.442%	0.328%
TCRBV07-09	3.2405%	2.8155%	3.526%	2.194%
TCRBV09-01	10.0821%	6.2841%	2.878%	1.680%
TCRBV10-01	0.4217%	0.5073%	0.253%	0.231%
TCRBV10-02	9.9046%	1.7945%	0.833%	1.233%
TCRBV10-03	1.2041%	0.9131%	1.090%	0.894%
TCRBV11	1.4149%	0.8687%	1.014%	0.700%
TCRBV11-01	0.2275%	0.1966%	0.120%	0.402%
TCRBV11-02	0.1165%	0.1395%	0.138%	0.082%
TCRBV11-03	0.3385%	0.4058%	0.319%	0.428%
TCRBV12	2.2694%	2.9930%	1.730%	4.641%
TCRBV12-01	0.0444%	0.0063%	0.011%	0.000%
TCRBV12-02	0.0277%	0.0634%	0.076%	0.071%
TCRBV12-03/12-04	3.3237%	3.1516%	2.125%	0.883%
TCRBV12-05	0.3052%	0.2093%	0.221%	0.164%
TCRBV13-01	1.1541%	1.8389%	0.778%	1.021%
TCRBV14-01	2.9519%	2.1560%	0.959%	0.373%
TCRBV15-01	0.9988%	0.7483%	0.634%	1.237%
TCRBV16-01	0.1665%	0.2156%	0.109%	0.045%

TCRBV18-01	1.4038%	1.7819%	1.220%	0.998%
TCRBV19-01	4.3891%	4.1725%	3.001%	3.039%
TCRBV20	4.2171%	5.1617%	5.083%	3.863%
TCRBV20-01	0.4772%	0.4566%	0.362%	0.298%
TCRBV21-01	0.6548%	0.3805%	0.261%	0.235%
TCRBV23-01	0.7269%	0.3614%	0.196%	0.075%
TCRBV24-01	1.3539%	1.7945%	1.238%	1.985%
TCRBV25	0.0111%	0.0000%	0.015%	0.007%
TCRBV25-01	0.5216%	0.3678%	0.395%	0.928%
TCRBV27-01	6.8416%	13.1706%	18.195%	18.568%
TCRBV28-01	3.7232%	4.6734%	13.554%	13.327%
TCRBV29-01	1.0154%	1.5409%	0.873%	0.354%
TCRBV30-01	1.0154%	0.7483%	0.500%	0.637%
TCRBVA-01	0.0111%	0.0000%	0.000%	0.000%

Supplementary Table 4C. TCRβJ family gene use in the CDR3 region of peripheral blood (PB) T cells before and 6 weeks after the first, second and third HER2 CAR T-cell infusions.

TCRβ J gene	PB pre-infusion	PB post-infusion 1	PB post-infusion 2	PB post-infusion 3
TCRBJ01-01	9.333	12.505	24.635	27.240
TCRBJ01-02	22.717	19.854	10.795	10.232
TCRBJ01-03	2.708	2.422	1.390	1.252
TCRBJ01-04	2.075	2.055	2.053	1.948
TCRBJ01-05	4.206	3.805	3.211	2.682
TCRBJ01-06	10.138	5.663	2.313	5.639
TCRBJ02	0.039	0.032	0.025	0.000
TCRBJ02-01	9.927	16.157	16.081	13.923
TCRBJ02-02	7.008	6.119	4.554	5.718
TCRBJ02-03	6.403	7.571	10.730	6.619
TCRBJ02-04	0.744	0.704	0.724	0.361
TCRBJ02-05	7.962	7.495	6.154	4.567
TCRBJ02-06	1.226	1.205	1.216	0.484
TCRBJ02-07	15.514	14.413	16.117	19.335

Supplementary Table 5A. List of hyperexpanded (>1% frequency) TCRβ CDR3 rearrangements present in peripheral blood (PB) during pre-infusion and their fate during the HER2 CAR T-cell induction period.

CDR3 sequence	Sum (Productive Frequency)	PB Pre-infusion	PB Infusion 1	PB Infusion 2	PB Infusion 3
CASSDDDSRYTF	10.674%	9.23%	1.40%	0.04%	0.01%
CASSVEGAENSPLHF	12.004%	7.43%	3.40%	0.84%	0.34%
CASSPRTSGGLTNTGELFF	3.957%	2.09%	0.83%	0.05%	0.98%
CASSQDRGNEQYF	3.717%	2.06%	1.22%	0.35%	0.07%
CASRVGQDYGYTF	6.333%	1.88%	2.45%	1.50%	0.50%
CASSLSPGPVGTQYF	2.332%	1.54%	0.66%	0.09%	0.04%
CASSSANYGYTF	1.979%	1.43%	0.46%	0.04%	0.04%

Supplementary Table 5B. Tracking of hyperexpanded (>1% frequency) TCRβ CDR3 rearrangements in peripheral blood (PB) during induction phase following HER2 CAR T-cell infusions.

CDR3 sequence	Sum (Productive Frequency)	PB Pre-infusion	PB Infusion 1	PB Infusion 2	PB Infusion 3
CASSLGGSYNEQFF	17.09%	0.90%	6.02%	7.35%	2.82%
CASSTPGQEGTDTQYF	12.86%	0.16%	2.21%	6.89%	3.62%
CASSGRDWDTVNTEAFF	6.40%	0.69%	2.09%	2.86%	0.76%
CASGWLTSSMNTEAFF	16.41%	0.00%	1.67%	9.28%	5.45%
CASSSANYGYTF	2.23%	0.60%	1.44%	0.04%	0.14%
CASSLLTTSEDYEQYF	8.94%	0.37%	1.40%	4.34%	2.83%
CASSFGGNDPRRYTF	2.29%	0.32%	1.20%	0.49%	0.28%
CASSLDHRELFF	1.84%	0.00%	1.14%	0.67%	0.02%
CASSYSSGGNTEAFF	12.38%	0.16%	0.35%	2.18%	9.70%
CASSGLSGETMNTEAFF	2.31%	0.03%	0.42%	1.69%	0.17%
CSARVPGLAGGTTGAQTQYF	3.10%	0.04%	0.54%	1.69%	0.84%
CASSGLEGTTMNTEAFF	5.72%	0.06%	0.06%	1.68%	3.93%
CASSSLPTGLAYEQYF	4.34%	0.01%	0.08%	1.63%	2.62%
CASSFGGTYNEQFF	2.37%	0.00%	0.62%	1.46%	0.30%
CASSELDTSLSYEQYF	3.88%	0.17%	0.49%	1.40%	1.83%
CASSLGLAPLHF	3.81%	0.00%	0.00%	0.01%	3.80%
CASSVRGNYGYTF	2.38%	0.00%	0.00%	0.00%	2.38%
CASSLGSGGALYGYTF	2.14%	0.00%	0.00%	0.00%	2.14%
CASRTSSNEQFF	1.80%	0.00%	0.00%	0.00%	1.80%
CASRTGPNEQYF	1.41%	0.00%	0.00%	0.00%	1.41%

Supplementary Table 6. Complete list of proteins eliciting increased signal from serum autoantibody binding (≥2 fold) in the patient's serum at various post HER2 CAR T-cell infusion time points (6 weeks after infusion 1, 2 and 3) compared to pre-infusion (4 weeks after standard chemotherapy but prior to study initiation) time point in Human Protein Microarray.

Data Base ID	Description	Gene symbol	Fold change from pre-infusion		
			Infusion 1 (Day 43)	Infusion 2 (Day 120)	Infusion 3 (Day 197)
BC002955.1	ubiquitin specific peptidase 2	*USP2*	27	11.3	6.1
NM_177403.3	RAB7B, member RAS oncogene family	*RAB7B*	10.7	8.1	4.2
NM_178154.1	fucosyltransferase 8 (alpha (1,6) fucosyl-transferase), transcript variant 2	*FUT8*	3.7	3.7	2.5
BC010895.1	sulfotransferase family, cytosolic, 1B, member 1	*SULT1B1*	2.2	2.9	2.8
BC017070.1	clusterin associated protein 1	*CLUAP1*		38.3	54.6
BC032347.1	chromosome 8 ORF 59	*C8ORF59*		12.1	6.4
	CCP_10BSA	*CCP-10BSA*		6.4	6.4
	CCP_1BSA	*CCP_1BSA*		5.2	4.2
NM_174972.1	CMP-N-acetylneuraminate-beta-1,4-galactoside alpha-2,3-sialyltransferase	*ST3GAL3*		3.8	6.9
NM_019884.2	glycogen synthase kinase 3 alpha	*GSK3A*	67.8	31.8	
XM_378678.1	PREDICTED: Homo sapiens hypothetical	*LOC400600*	27.2	12.4	
NM_016484.1	PDZ domain containing 11	*PDZD11*	18.6		
BC025279.1	Scaffold attachment factor	*SAFB2*	9.4		
NM_001023.2	ribosomal protein S20	*RPS20*	8.6		
NM_001896.1	Casein kinase 2, alpha prime polypeptide, mRNA	*CSNK2A2*	6		
BC014271.2	endoglin (Osler-Rendu-Weber syndrome 1)	*ENG*	5.5		
NM_001025100.1	Myelin basic protein	*MBP*	5.4		
BC033219.1	Leucine zipper protein 1, mRNA (cDNA clone IMAGE:5017908) complete cds	*LUZP1*	4.8		
NP_002497.2	B-NGF / Beta-NGF Protein (Native)	*NGF*	4.3		
BC010629.1	outer dense fiber of sperm tails 2	*ODF2*	3.4		
NM_024695.1	lectin, mannose-binding, 1 like	*LMAN1L*	3.3		
BC012109.1	homer scaffolding protein 2	*HOMER2*	3		
NM_006917.3	Retinoic acid receptor RXR-gamma	*RXRG*	2.5		
NM_002055.1	glial fibrillary acidic protein	*GFAP*	2.2		
NM_015640.1	SERPINE1 mRNA binding protein 1, transcript variant 4	*SERBP1*	2.2		
NM_016359.2	Nucleolar and spindle associated protein 1, transcript variant 1, mRNA	*NUSAP1*		9.7	
NM_001003396.1	tumor protein D52-like 1, transcript variant 3	*TPD52L1*		4.8	
NM_015975.3	TAF9B RNA polymerase II, TATA box binding protein (TBP)-associated factor, 31kDa	*TAF9B*		4.3	
NM_002608.1	platelet-derived growth factor beta polypeptide (simian sarcoma viral (v-sis) oncogene homolog) (PDGFB), transcript variant 1	*PDGFB*		2.3	
NM_005898.4	cell cycle associated protein 1, transcript variant 1	*CAPRIN1*		2.1	
NM_012325.1	microtubule-associated protein, RP/EB family, member 1	*MAPRE1*		2	
NM_199328.1	Claudin 8	*CLDN8*		2	
NM_173191.2	Kv channel interacting protein 2, transcript variant 2	*KCNIP2*			223.3
NM_006147.1	interferon regulatory factor 6	*IRF6*			168
NM_018107.2	RNA binding motif protein 23, transcript variant 2	*RBM23*			97.6

BC024208.1	RNA binding motif protein 23	*RBM23*			73.1
BC009348.2	cirrhosis, autosomal recessive 1A (cirhin)	*CIRH1A*			9.7
NM_153207.2	AE binding protein 2	*AEBP2*			9.1
BC052813.1	Excision repair cross-complementing rodent repair deficiency, complementation group 1	*ERCC1*			7.1
NM_199124.1	chromosome 11 open reading frame 63 (C11orf63), transcript variant 2	*C11orf63*			5.6
BC080187.1	Leiomodin-1	*LMOD-1*			4.1
BC001755.1	Leiomodin-1	*LMOD-1*			3.7

Supplementary Table 7. Proteins consistently eliciting increased signal from serum autoantibody binding (≥2 fold) detected 6 weeks after the first, second and third infusions, their localization, and pathology association.

Gene ID	Description	Cellular Localization[1,2]	Pathology Association
FUT8	Fucosyltransferase 8 (alpha (1,6) fucosyl-transferase), transcript variant 2	Cytosol, plasma membrane	Promotes breast cancer cell invasiveness[3], melanoma metastasis[4], overexpressed in aggressive prostate cancer[5]
USP2	Ubiquitin specific peptidase 2	Golgi apparatus, nucleoplasm	Promotes cell migration and invasion in Triple negative breast cancer[6], colorectal cancer and mantle cell lymphoma models[7], enhances tumor progression in bladder cancer[8]
SULT1B1	Sulfotransferase family, cytosolic, 1B, member 1	Cytosol, Golgi apparatus, plasma membrane	Expressed in colorectal cancer with copy number aberrations[9]
RAB7B	*RAB7B* member, *RAS* oncogene family	Cytosol	Unfavorable prognostic marker in endometrial cancer[1], monocytic differentiation of human acute promyelocytic leukemia cells[10], interacts directly with myosin II and influences cell adhesion, polarization and migration[11]

Supplementary References

1. Uhlen M, Zhang C, Lee S, et al. A pathology atlas of the human cancer transcriptome. Science 2017;357.

2. UniProt Consortium T. UniProt: the universal protein knowledgebase. Nucleic acids research 2018;46:2699.

3. Tu CF, Wu MY, Lin YC, Kannagi R, Yang RB. FUT8 promotes breast cancer cell invasiveness by remodeling TGF-beta receptor core fucosylation. Breast cancer research : BCR 2017;19:111.

4. Agrawal P, Fontanals-Cirera B, Sokolova E, et al. A Systems Biology Approach Identifies FUT8 as a Driver of Melanoma Metastasis. Cancer cell 2017;31:804-19 e7.

5. Wang X, Chen J, Li QK, et al. Overexpression of alpha (1,6) fucosyltransferase associated with aggressive prostate cancer. Glycobiology 2014;24:935-44.

6. Qu Q, Mao Y, Xiao G, et al. USP2 promotes cell migration and invasion in triple negative breast cancer cell lines. Tumour biology : the journal of the International Society for Oncodevelopmental Biology and Medicine 2015;36:5415-23.

7. Davis MI, Pragani R, Fox JT, et al. Small Molecule Inhibition of the Ubiquitin-specific Protease USP2 Accelerates cyclin D1 Degradation and Leads to Cell Cycle Arrest in Colorectal Cancer and Mantle Cell Lymphoma Models. The Journal of biological chemistry 2016;291:24628-40.

8. Kim J, Kim WJ, Liu Z, Loda M, Freeman MR. The ubiquitin-specific protease USP2a enhances tumor progression by targeting cyclin A1 in bladder cancer. Cell cycle 2012;11:1123-30.

9. Yoshida T, Kobayashi T, Itoda M, et al. Clinical omics analysis of colorectal cancer incorporating copy number aberrations and gene expression data. Cancer informatics 2010;9:147-61.

10. Yang M, Chen T, Han C, Li N, Wan T, Cao X. Rab7b, a novel lysosome-associated small GTPase, is involved in monocytic differentiation of human acute promyelocytic leukemia cells. Biochemical and biophysical research communications 2004;318:792-9.

11. Borg M, Bakke O, Progida C. A novel interaction between Rab7b and actomyosin reveals a dual role in intracellular transport and cell migration. Journal of cell science 2014;127:4927-39.
