# Supplementary material for: Tumor response and endogenous immune reactivity after administration of HER2 CAR T cells in a child with metastatic rhabdomyosarcoma
Source: Nat Commun. 2020 Jul 15;11:3549. doi: 10.1038/s41467-020-17175-8 (PMC7363864; doi:10.1038/s41467-020-17175-8)
Supplement: Supplementary file 2 — Reporting Summary [file 41467_2020_17175_MOESM2_ESM.pdf]

natureresearch

Corresponding author(s): Meenakshi Hegde
Nabil Ahmed

Last updated by author(s): 5/14/2020

Reporting Summary

Nature Research wishes to improve the reproducibility of the work that we publish. This form provides structure for consistency and transparency in reporting. For further information on Nature Research policies, seeAuthors & Referees and theEditorial Policy Checklist .

Statistics

For all statistical analyses, confirm that the following items are present in the figure legend, table legend, main text, or Methods section.

n/a	Confirmed	
☐	☒	The exact sample size (*n*) for each experimental group/condition, given as a discrete number and unit of measurement
☐	☒	A statement on whether measurements were taken from distinct samples or whether the same sample was measured repeatedly
☒	☐	The statistical test(s) used AND whether they are one- or two-sided<br>*Only common tests should be described solely by name; describe more complex techniques in the Methods section.*
☒	☐	A description of all covariates tested
☒	☐	A description of any assumptions or corrections, such as tests of normality and adjustment for multiple comparisons
☐	☒	A full description of the statistical parameters including central tendency (e.g. means) or other basic estimates (e.g. regression coefficient) AND variation (e.g. standard deviation) or associated estimates of uncertainty (e.g. confidence intervals)
☒	☐	For null hypothesis testing, the test statistic (e.g. *F*, *t*, *r*) with confidence intervals, effect sizes, degrees of freedom and *P* value noted<br>*Give P values as exact values whenever suitable.*
☒	☐	For Bayesian analysis, information on the choice of priors and Markov chain Monte Carlo settings
☒	☐	For hierarchical and complex designs, identification of the appropriate level for tests and full reporting of outcomes
☒	☐	Estimates of effect sizes (e.g. Cohen's *d*, Pearson's *r*), indicating how they were calculated

Our web collection on statistics for biologists contains articles on many of the points above.

Software and code

Policy information about availability of computer code

Data collection	Flow data - BD Accuri C6 flow cytometer/software and Gallios Flow cytometer with Kaluza software; qPCR - ABI 7900HT Sequence Detection System (Applied Biosystems), ELISA - Infinite® F50 microplate reader with Magellan software. No custom codes were used in the study.
Data analysis	FlowJo v10 for all flow cytometry analysis, Prism 8.0, Microsoft Excel 2013, ImmunoSEQAnalyzer 3.0 software for analysis of TCRbeta sequencing, ProtoArray Prospector v5.2.3, WebGIVI tool (http://raven.anr.udel.edu/webgivi/) for creating cytoscape maps, webtool at http://bioinformatics.psb.ugent.be/webtools/Venn/ for creating Venn plots.

For manuscripts utilizing custom algorithms or software that are central to the research but not yet described in published literature, software must be made available to editors/reviewers. We strongly encourage code deposition in a community repository (e.g. GitHub). See the Nature Research guidelines for submitting code & software for further information.

Data

Policy information about availability of data

All manuscripts must include a data availability statement. This statement should provide the following information, where applicable:

- Accession codes, unique identifiers, or web links for publicly available datasets
- A list of figures that have associated raw data
- A description of any restrictions on data availability

Data Availability Statement is included in the manuscript. The source data underlying Figures 1(F), 2(A-E), 3(D-G), 4(A-D), 5(A-E), 6(A-C), 7(C, D, F-I) and Supplementary Figures 1(A-D), 2(A-E), 3(A-B) are provided as a Source Data file. ProtoArrayTM data in this publication is deposited in NCBI's functional genomic data repository Gene Expression Omnibus (GEO) and is accessible through GEO series accession number GSE152029. Figures 1G and 7E are created by Shoba Navai, MD, using BioRender.com (purchased license). For these Figures, timeline from initial study enrollment is provided without the dates to protect the research participant's privacy, and to align with the study consent. All the other data supporting the findings of this study are available within the article, supplementary information, source files and from the corresponding author upon reasonable request.

Field-specific reporting

Please select the one below that is the best fit for your research. If you are not sure, read the appropriate sections before making your selection.

☒ Life sciences ☐ Behavioural & social sciences ☐ Ecological, evolutionary & environmental sciences

For a reference copy of the document with all sections, see nature.com/documents/nr-reporting-summary-flat.pdf

Life sciences study design

All studies must disclose on these points even when the disclosure is negative.

Sample size	The phase I study is ongoing and this is a single patient report describing the exceptional clinical outcome and evidence of endogenous immune reactivity. All clinical laboratory studies were done in CLIA certified laboratories following the standard testing methods. For immune correlative studies carried out the in the laboratory, two or more biologically independent samples (e.g. serum samples collected at different time points) were analyzed over time and technical replicates (2 or 3 as appropriate) were used when indicated in a given experiment. CAR T-cell product is evaluated for transduction efficiency at two independent time points before product release. Disease evaluation with histopathological examination of the bone marrow and whole-body PET-CT were done as part of patient care following standard clinical guidelines. Representative images are provided for these evaluations.
Data exclusions	All relevant clinical and laboratory data available are reported. No data were excluded from the report.
Replication	CAR T-cell product is evaluated for transduction efficiency using flow cytometry at two independent time points before product release. Phenotype analysis is done by the GMP facility using an established experimental method for characterizing all T-cell products manufactured. CAR T-cell product is tested for cytolytic activity using two HER2-positive and two HER2-negative tumor cell lines at 4 effector to target ratios (5:1, 10:1, 20:1, 40:1). Representative graphs in provided depict the cytolytic activity against one HER2-positive and one HER2-negative tumor target at 20:1 effector to target ratio. Disease evaluation with histopathological examination of the bone marrow and whole-body PET-CT were done as part of patient care following standard clinical guidelines. Representative images are provided for these evaluations. Post-infusion flow analysis of the PBMCs for detecting CAR T cells and evaluating expression of PD1/LAG3 were done as two independent experiments using HER2Fc+anti-human Fc and anti-F(ab) antibody respectively for detecting CAR expressing T cells. Keeping the limited patient samples in mind, both of these flow experiments were optimized and repeated using donor PBMC containing HER2 CAR T cells at decreasing concentrations prior to testing patient PBMCs. Indirect ELISA for rGSK3A and total IgG/IgM levels were performed as technical replicates (2 or 3 as appropriate), each in a single experiment at different dilutions (specified in the manuscript text). Serum cytokines were analysed using the Luminex multiplex kit in two independent experiments with 2 technical replicates for each biologically distinct sample. qPCR for detecting CAR T cells in the peripheral blood was done as an independent experiment for each time point by the GLP facility, using the same experimental method (described in the manuscript text) for patients enrolled on all clinical trials of FRP5-based HER2 CAR T cells at our institution.
Randomization	Not relevant as this is a single patient report from a Phase I study.
Blinding	This is a Phase 1 open label study. Protoarray analysis (Thermofisher Scientific) and TCR-beta survey sequencing (Adaptive Biotechnologies) were performed as pay for service on coded samples and decoded during analysis.

Reporting for specific materials, systems and methods

We require information from authors about some types of materials, experimental systems and methods used in many studies. Here, indicate whether each material, system or method listed is relevant to your study. If you are not sure if a list item applies to your research, read the appropriate section before selecting a response.

Materials & experimental systems

n/a	Involved in the study	
☐	☒	Antibodies
☐	☒	Eukaryotic cell lines
☒	☐	Palaeontology
☒	☐	Animals and other organisms
☐	☒	Human research participants
☐	☒	Clinical data

Methods

n/a	Involved in the study	
☒	☐	ChIP-seq
☐	☒	Flow cytometry
☒	☐	MRI-based neuroimaging

Antibodies

Antibodies used	The following monoclonal antibodies were used: mouse anti-human CD3 FITC (clone: SK7, Cat #: 349201, Lot #: 7221481, Becton Dickinson Biosciences, San Jose, CA), mouse anti-human CD8 Pacific Blue (clone: B9.11, Cat #: A82791, Lot #: 38, Beckman Coulter, Indianapolis, IN), Mouse anti-Human CD279 (PD-1) APC (clone: MIH4, Cat #: 558694, Lot #: 8012764, Becton Dickinson Biosciences, San Jose, CA), mouse anti-human CD223 (LAG-3) FITC (clone: 3DS223H, Cat#: 11-2239-42, Lot #: 4337363, Thermo

Fisher Scientific, Waltham, MA). HER2-CAR expression was detected with either: Alexa Fluor® 647 AffiniPure Goat Anti-Mouse IgG, F(ab')2 fragment specific (polyclonal, Cat #: 115-605-006, Lot #: 122475, The Jackson Laboratory, Bar Harbor, ME), or a recombinant Human ErbB2/Her2 Fc chimera protein (R&D Systems, Cat #: 1129-ER, Lot #: FXR0917071, Minneapolis, MN) followed by a goat anti-human IgG Fc secondary antibody PE (polyclonal, Cat #: 12-4998-82, Lot #: 2070588, Thermo Fisher Scientific, Waltham, MA). Negative controls included isotype antibodies. All flow antibodies were used in 1:20 dilution (stock solution, 5 uL/100 uL test condition) except for goat anti-human IgG Fc secondary antibody PE (pre-titrated) which was used in 1:100 dilution (1uL/100uL test condition) and F(ab)2-fragment specific antibody which was reconstituted at 1:100 to 1:800 dilution and used at 0.5-1 uL per 100 uL test condition. For HER2 immunohistochemistry, anti-ErbB2 antibody, clone CB11, Abcam, Cat#ab8054. For ELISA: IgG (total) Human uncoated ELISA kit (Cat# 88-50550-22, Lot# 175941117) and IgM Human uncoated ELISA kit (Cat# 88-50620-22, Lot# 1666010115); indirect ELISA for detecting serum reactivity to oncogenic pathway proteins, goat anti-human IgG (γ-chain specific) conjugated to HRP (1:2500 dilution; Cat# A8419-2ML, Lot# 077M4873V, Sigma-Aldrich, St. Louis, MO) was used as secondary antibody.

Validation

Clinical product testing and phenotyping were performed in the FACT accredited GMP / CLIA certified GLP facility at Texas Children's Hospital. All antibodies used were commercially available and validated by the manufacturer for specificity and function. Due to reference limitations, clone information and the catalogue numbers are provided in the manuscript for each of these to enable detailed validation information upon online search.

Eukaryotic cell lines

Policy information about cell lines

Cell line source(s)

MDA-MB-468, NCI-H1299, LM7 and K562 were used for clinical product testing. Cell lines were obtained from ATCC.

Authentication

Cell lines were authenticated using short tandem repeat (STR) analysis.

Mycoplasma contamination

The cell lines were mycoplasma negative.

Commonly misidentified lines (See ICLAC register)

None identified in the ICLAC register.

Human research participants

Policy information about studies involving human research participants

Population characteristics

Human subject research participants were eligible for study enrollment if they had a recurrent or progressive HER2-positive sarcoma that had failed at least one line of standard of care treatment. There was no defined age limit or gender limitation to the study. Participants were required to meet organ function criteria as defined in the clinical trial protocol. Majority of the participants where children and young adults as expected given the age distribution of bone and soft tissue sarcomas.

Recruitment

The trial was posted publicly to www.clinicaltrials.gov (NCT00902044). Referrals to the study were either self-driven (patient/parents) or from the patient's primary oncologist. This participant was referred to our phase I trial by his primary oncologist at an independent institution making the possibility of self-selection bias unlikely.

Ethics oversight

The study was approved by the Institutional Review Board (IRB) of Baylor College of Medicine, Texas Children's Hospital, and Houston Methodist Hospital. The study protocol was also approved by the U.S. Food and Drug Administration (FDA) and the Recombinant DNA Advisory Committee (RAC) of the National Institutes of Health (NIH). A statement of participant consent is included in the manuscript text.

Note that full information on the approval of the study protocol must also be provided in the manuscript.

Clinical data

Policy information about clinical studies

All manuscripts should comply with the ICMJEguidelines for publication of clinical research and a completedCONSORT checklist must be included with all submissions.

Clinical trial registration

NCT00902044

Study protocol

The full trial protocol has been provided to reviewers/editors and is available from the corresponding author upon reasonable request.

Data collection

The lymphodepletion cohort of this trial has been open to enrollment since March 2015 and is currently ongoing. Clinical and laboratory evaluations are performed as per the study evaluation calendar. Data collection was done by the assigned study coordinator. Data specified in the clinical trial protocol were collected at pre-determined time points: during the hospitalization for receiving treatment and follow up study visits to the outpatient clinic. T-cell infusions were given in the Texas Children's Hospital bone marrow transplant unit. Outpatient follow ups were done at the Texas Children's Hospital Clinical Research Center (CRC) and Yale New Haven Children's Hospital. Providing these specifics in the manuscript text describing a single patient could compromise patient privacy and hence not included.

Outcomes

The primary objective of this Phase I study is to determine the safety of autologous HER2 CAR T cells infused after lymphodepleting chemotherapy. Toxicities were evaluated using the NCI CTCAE version 4, with the exception of cytokine release syndrome (CRS) and neurological toxicities which were graded according to published guidelines specified in the clinical trial protocol (Lee et al). Initial safety evaluation period is 6 weeks after each T cell infusion. Safety and toxicity outcomes are

measured and reported to the FDA in real time. Disease evaluation was performed with whole-body PET-CT and bilateral BMAB prior to study entry and at 6 weeks after each T-cell infusion to assess the treatment response. Histologic BMAB response was tested and interpreted by an independent clinical pathologist per standard of care. Radiographic responses were evaluated using Response Evaluation Criteria in Solid Tumors (RECIST).

Flow Cytometry

Plots

Confirm that:

- [x] The axis labels state the marker and fluorochrome used (e.g. CD4-FITC).
- [x] The axis scales are clearly visible. Include numbers along axes only for bottom left plot of group (a 'group' is an analysis of identical markers).
- [x] All plots are contour plots with outliers or pseudocolor plots.
- [x] A numerical value for number of cells or percentage (with statistics) is provided.

Methodology

Sample preparation	0.5-1x10E6 cells were aliquoted and washed twice with 3ml PBS by centrifugation. Cells were resuspended in 100 ul of diluted primary antibody (prepared in PBS at the specified dilution) and incubated for 1 hour at 4c in the dark. Cells were then washed in 3ml PBS by centrifugation. Cells were resuspended in 100 ul of PBS and analyzed on a flow cytometer. For HER2 staining using the HER2-Fc chimeric protein, cells were first incubated with HER2-Fc protein and then washed once in 3ml PBS by centrifugation. Cells were resuspended in 100 ul and incubated with the secondary anti-human Fc antibody for 1 hour at 4c in the dark. Cells were then washed in 3ml PBS by centrifugation. Cells were resuspended in 100 ul of PBS and analyzed on a flow cytometer.
Instrument	BD ACCURI C6 (Becton Dickinson); Gallios flow cytometer (Beckman Coulter Inc.)
Software	FlowJo v10.4; Gallios cytometer v1.1; BD Accuri C6 software
Cell population abundance	Figure 3C: for PBMC post-infusion2 sample; CD3+ cells: 66.4%, HER2 CAR+ cells: 2.21%.<br>Figure 3D & E: for PBMC post-infusion1 sample: CD3+ cells: 53%, HER2 CAR+ cells: 1.98%. for PBMC post-infusion2 sample; CD3+ cells: 66.4%, HER2 CAR+ cells: 2.21%. for PBMC post-infusion3 sample; CD3+ cells: 63.4%, HER2 CAR+ cells: 1.43%.
Gating strategy	Gating strategies for all flow cytometry analysis are provided in the supplementary information.<br>Figure 2B: The samples were plotted on SSC vs FSC to gate on live lymphocytes. Live lymphocytes were plotted on a histogram with HER2 CAR-Alexa Flour 647 on the X-axis. The gate for Alexa Flour 647 positive cells was based on control non-transduced (NT) cells that was treated in parallel with the CAR T cell product sample.<br>Figure 3C: The samples were plotted on SSC vs FSC to gate on live lymphocytes. Live lymphocytes were plotted on SCC vs CD3-FITC. CD3 positive cells were plotted on SSC vs HER2 CAR-PE (stained with HER2-Fc then secondary anti-human Fc-PE antibody). The gate for HER2 CAR-PE positive cells was based on control non-transduced (NT) cells that was run in parallel.

- [x] Tick this box to confirm that a figure exemplifying the gating strategy is provided in the Supplementary Information.
